# ﻿From seagrass roots to saline soils: discovery of two new genera in *Lulworthiales* (*Sordariomycetes*) from osmotically stressed habitats

**DOI:** 10.3897/imafungus.16.157688

**Published:** 2025-08-12

**Authors:** Martina Réblová, Jana Nekvindová, Ondřej Hynar, Martin Vohník

**Affiliations:** 1 Department of Taxonomy, Institute of Botany, Czech Academy of Sciences, Zámek 1, Průhonice, Czech Republic; 2 Institute of Clinical Biochemistry and Diagnostics, University Hospital Hradec Králové, Sokolská 581, Hradec Králové, Czech Republic; 3 Antonína Slavíka 1311/4, Brno, Czech Republic; 4 Department of Mycorrhizal Symbioses, Institute of Botany, Czech Academy of Sciences, Lesní 322, Průhonice, Czech Republic; 5 KROKODIVE.CZ, Údolní 219/47, Prague, Czech Republic

**Keywords:** Dictyoconidia, holoblastic conidiogenesis, marine, monilioid, new taxa, phylogenetics, saprobic, symbiotic, *Thalassodendron*

## Abstract

As part of an ongoing study of marine fungi associated with seagrasses, we discovered a novel root-fungus symbiosis in the Indo-Pacific species *Thalassodendronciliatum* from Mauritius. Culturing its mycobionts yielded dozens of morphologically and genetically uniform isolates, all representing a previously unknown fungus. A second undescribed fungus was isolated from saline soils in Czechia. Phylogenetic analyses based on three rDNA markers confirmed both taxa as distinct, hitherto unknown lineages within the *Lulworthiales*, which are introduced here as *Thalassodendromycespurpureus***gen. et sp. nov.** and *Halomyrmapluriseptata***gen. et sp. nov.**, respectively. Both species developed characteristic structures under culture conditions that enabled their morphological characterisation: *T.purpureus* forms distinctive clusters of dark brown monilioid hyphae, while *H.pluriseptata* is characterised by holoblastic conidiogenesis and solitary, dark brown, multicellular conidia. *Thalassodendromyces* clustered in a strongly supported clade with *Spathulospora*, a parasitic genus of the red macroalga *Ballia*, while the closest relatives of *Halomyrma* were identified as the asexual genera *Halazoon* and *Halophilomyces* (nom. inval. Art. 40.7). An analysis of published metabarcoding ITS rDNA data from environmental samples in the GlobalFungi database indicated that *H.pluriseptata* is widely distributed across temperate, subtropical, and tropical regions in the Northern and Southern Hemispheres. The species exhibits a strong preference for aquatic biomes, particularly marine and estuarine, with a few records in terrestrial ecosystems. In contrast, no record of *T.purpureus* was retrieved from GlobalFungi, suggesting narrower ecological specialisation, a close association with its seagrass host, and/or a restricted geographical range. Our findings expand the ecological and phylogenetic scope of the *Lulworthiales*, bridging marine and terrestrial fungal communities, and highlight seagrass roots as an important source of novel symbiotic marine fungi. Recent discoveries of the *Lulworthiales* in saline inland soils challenge their marine exclusivity and raise important questions about their ecological plasticity, dispersal mechanisms, and adaptive strategies. In light of current observations, we discuss the taxonomic challenges of the *Spathulosporales* and the lulworthialean fungi, integrating molecular and morphological perspectives. We address the importance of combining morphological and molecular approaches to accurately delineate new fungal taxa, as well as the value of environmental DNA metabarcoding for uncovering cryptic fungal diversity and enhancing our understanding of fungal distribution and ecological functions.

## ﻿Introduction

Marine fungi represent an ecologically diverse group of microorganisms that inhabit a wide range of environments, including seawater, sea foam, corals, driftwood, sediments, seagrasses and seaweeds, plants in intertidal zones, decaying organic matter, and even the exoskeletons of marine animals. They play critical roles in nutrient cycling and organic matter decomposition, significantly contributing to the stability and function of marine ecosystem dynamics (e.g. Johnson and Sparrow 1961; Kohlmeyer and Kohlmeyer 1979; Kohlmeyer and Volkmann-Kohlmeyer 1991; Jones 2011; Richards et al. 2012; Jones et al. 2015; Kumar et al. 2021; Gonçalves et al. 2022). Among the various groups of marine fungi, the order *Lulworthiales* (Kohlmeyer et al. 2000) holds ecological and taxonomic importance due to its specialisation in brackish and saline habitats, ranging from coastal to deep-sea ecosystems.

A comprehensive treatment on the morphology and phylogenetic relationships of the members of *Lulworthiales* was provided by Dayarathne et al. (2025). The *Lulworthiales* currently comprise 21 genera (MycoBank, Crous et al. 2004), 12 of which are known to produce sexual morphs. These include *Haloguignardia* (Cribb and Cribb 1956), *Kohlmeyeriella* (Jones et al. 1983), Lindra (Li.) (Wilson 1956), *Lindriella* (Dayarathne et al. 2025), *Lulwoidea* (Campbell et al. 2005), Lulworthia (L.) (Sutherland 1915), *Paralulworthia* (Poli et al. 2020), *Rambellisea* (Braconcini et al. 2024), *Rostrupiella* (Koch et al. 2007), *Sammeyersia* (Abdel-Wahab et al. 2017), *Spathulospora* (Cavaliere and Johnson 1965), and *Zalerion* (syn. Lulwoana (Lu.), Moore and Meyers 1962; Gonçalves et al. 2021). They are characterised by perithecial, non-stromatic ascomata lacking a hamathecium, unitunicate early deliquescing asci, and filamentous, hyaline ascospores that may or may not exhibit apical chambers or appendages. However, only two of them, namely *Lindra* and *Zalerion*, have been demonstrated to be holomorphic through experimental verification of their complete life cycles (Nakagiri and Tubaki 1983; Nakagiri 1984). The genera lacking an observed sexual state comprise *Cumulospora* (Schmidt 1985), *Halazoon*, *Hydea*, *Matsusporium*, *Moleospora*, *Moromyces* (Abdel-Wahab et al. 2010), *Orbimyces* (Barghoorn and Linder 1944), and *Paramoleospora* (Li et al. 2023). *Halophilomyces* (Wang et al. 2024), described for asexually reproducing fungi, is currently considered an invalid genus name under Article 40.7 of the International Code of Nomenclature (ICN) (Klopper et al. 2025). Members of these genera are characterised by holoblastic conidiogenesis and pigmented conidia that range from ellipsoidal, curved, or helicoid forms to sometimes multicellular structures (dictyoconidia). The clusters, or chain-like aggregations of globose cells, easily separate under slight pressure, while the solitary globose cells are ornamented with a crown-like arrangement of appendages. In some species, the conidia may be hyaline and filiform.

*Spathulospora*, typified by *S.phycophila*, was initially placed in its own order, *Spathulosporales* (Kohlmeyer 1973), given its parasitic lifestyle and lack of hyphal growth. However, phylogenetic analyses based on partial nuclear small subunit (SSU) and large subunit (LSU) rDNA sequences obtained from dried herbarium specimens of *S.adelpha* and *S.antarctica* revealed that the genus is nested within the *Lulworthiales* (Inderbitzin et al. 2004), a finding that has been subsequently corroborated by further molecular studies. The *Lulworthiales* are classified within the subclass *Lulworthiomycetidae (Sordariomycetes)*, alongside their closest relatives in the order *Koralionastetales* (Kohlmeyer and Volkmann-Kohlmeyer 1989; Campbell et al. 2009). The latter comprises two genera, *Koralionastes*, commonly isolated from coral substrates, and *Pontogeneia*, a parasite of brown macroalgae (Kohlmeyer 1975; Kohlmeyer and Volkman-Kohlmeyer 1987a).

Members of the *Lulworthiales* thrive in areas of constant or periodic seawater immersion, typically in intertidal and subtidal zones, including mangroves, estuaries, and salt marshes. They primarily specialise in degradation of submerged substrates by breaking down organic material and facilitating nutrient turnover, colonising submerged decaying wood, such as untreated or partially decayed pilings and wood bait panels submerged in seawater, driftwood, and herbaceous stems of intertidal angiosperms (e.g. Wilson 1956; Johnson 1958; Kohlmeyer and Volkmann-Kohlmeyer 1987b; Kohlmeyer et al. 2000; Koch et al. 2007; Jones et al. 2008; Abdel-Wahab et al. 2010; Azevedo et al. 2017). They inhabit leaves, roots, and rhizomes of seagrasses (e.g. Orpurt et al. 1964; Meyers 1969; Cuomo et al. 1985; Hyde and Jones 1989; Poli et al. 2020, 2021; Vohník 2022) and are also found on macroalgal thalli, commonly known as seaweeds (Estee 1913; Sutherland 1915; Cavaliere and Johnson 1965; Meyers 1969; Kohlmeyer 1973, 1984; Kohlmeyer and Volkmann-Kohlmeyer 2003). Unique habitats include the exoskeletons of marine tunicates (Braconcini et al. 2024) and decaying animal substrates such as feathers, calcareous shells, and cuttlebone (Nambiar and Raveendran 2015). Additionally, some species exhibit an arenicolous lifestyle (Koch and Jones 1984; Nakagiri 1984; Pang et al. 2023). They colonise driftwood buried in sand, but their ascomata form directly among the sand grains, connected to the original wood substrate only by mycelium. Other unusual biotopes include coral reefs (Kohlmeyer 1984; Kohlmeyer and Volkman-Kohlmeyer 1989; unpublished record: GenBank KU359242, KU359244) and sea foam on sandy beaches, which serves as a natural trap for debris, small marine organisms, and fungal spores, including ascospores and conidia of several lulworthialean species (Nakagiri and Tubaki 1983; Nakagiri 1989). This environment provides a unique dispersal mechanism for marine fungi, facilitating their spread across coastal ecosystems. Although the *Lulworthiales* have traditionally been linked to marine and estuarine environments, recent environmental DNA (eDNA) metabarcoding studies indicate their presence in fungal communities in both coastal and inland soil ecosystems (Li et al. 2019; Xiao et al. 2020). This hypothesis is further supported by two cultivable lulworthialean fungi reported from these habitats: *Lulwoana* sp. from saline inland soils (Hujslová et al. 2010) and *Paramoleospora* from mangrove sediments (Li et al. 2023).

The *Lulworthiales* are globally distributed, ranging from polar to tropical regions, wherever suitable substrates are available. On the other hand, the geographic distribution of some genera seems to be limited, as exemplified by *Spathulospora*, which is exclusive to the Atlantic, Pacific, and Indian Oceans of the Southern Hemisphere (Kohlmeyer 1973). Available records indicate that lulworthialean fungi have been extensively studied in tropical, subtropical, and temperate regions, whereas they remain largely unexplored in polar environments (Rämä et al. 2014; Hagestad et al. 2020; Tibell et al. 2020).

Members of the order *Lulworthiales* occupy a diverse range of ecological niches, and although they are predominantly saprobic, some species adopt a parasitic lifestyle. Species of *Spathulospora* (Cavaliere and Johnson 1965; Kohlmeyer 1973) were introduced as parasites of the red alga *Ballia*. The nature of this association was elucidated by Walker et al. (1979), who identified *Spathulospora* as a biotrophic parasite. *Lindrathalassiae* was isolated from necrotic leaves of the seagrass *Thalassiatestudinum*, in what appeared to be infected leaf tissue exhibiting necrotic symptoms resembling those seen on terrestrial grasses affected by various fungal pathogens (Orpurt et al. 1964; Meyers 1965). More recently, Braconcini et al. (2024) introduced the genus *Rambellisea*, an epizoic fungus associated with the marine tunicate *Halocynthiapapillosa* (*Ascidiacea*). Remarkably, the tunic of this organism partially consists of cellulose, a biomolecule exceptionally rare in animals.

The leaves, roots, and rhizomes of seagrasses represent specialised ecological niches, providing habitat for diverse fungal communities. Seagrasses are flowering plants that grow partially or fully submerged in shallow coastal waters, often forming dense underwater meadows, and whose pollination takes place in seawater. They occur globally, spanning from tropical to subarctic regions, and play a vital role in marine ecosystems. The fungal endophyte communities associated with several seagrass species have been extensively explored, either via culture-based or high-throughput sequencing studies, most notably in *Cymodocea*, *Enhalus*, *Halodule*, *Halophila*, *Posidonia*, *Thalassia*, and *Zostera* (e.g. Devarajan et al. 2002; Sakayaroj et al. 2010; Panno et al. 2011; Mata and Cebrián 2013; Shoemaker and Wyllie-Echeverria 2013; Gnavi et al. 2014; Supaphon et al. 2017; Torta et al. 2015, 2022; Venkatachalam et al. 2015; Vohník et al. 2016, 2017, 2019; Ettinger and Eisen 2020; Pasqualetti et al. 2020; Poli et al. 2020; Abdel-Wahab et al. 2021; Kinamot and Monotilla 2023; Tasdemir et al. 2024; Ugarellia et al. 2024). Rajakaruna et al. (2024) highlighted the biotechnological and pharmaceutical potential of seagrass-associated endophytic fungi, further emphasising the need to explore their diversity and ecological roles. To date, only a few lulworthialean fungi have been documented within the living tissues of seagrasses (e.g. Orpurt et al. 1964; Newell 1981; Cuomo et al. 1985; Hyde and Jones 1989; Poli et al. 2020, 2021; Vohník 2021, 2022). Although many have not been identified to species level and are reported as *Lulwoana* sp., *Lulworthia* sp., or as undescribed dark septate (DS) endophytes (Torta et al. 2015, 2022; Vohník et al. 2017; Vohník 2022), current knowledge of lulworthialean endophytes in seagrasses is confined to *Paralulworthia* (Poli et al. 2020, 2021) and *Halophilomyces* (Wang et al. 2024).

Inland saline habitats, such as salt marshes, coastal lagoons, saline lakes, saline deserts, and soils with varying degrees of salinity, represent rare and extreme environments where fungal diversity and adaptive mechanisms remain largely unexplored. Studies have suggested that fungi in these environments must develop specialised adaptations to cope with osmotic stress and fluctuating salinity levels (e.g. Cantrell et al. 2006; Butinar et al. 2011).

To tackle various challenges associated with marine fungi, integrating morphological, molecular, biogeographic, and physiological studies of culturable representatives can help refine our understanding of the *Lulworthiales*. Furthermore, examining the distribution of these fungi across marine and terrestrial biomes helps to uncover potential pathways of dispersal, whether through airborne spores, ocean currents, or plant-mediated transmission. Assessing their global occurrence also contributes to predicting their responses to environmental changes, such as increasing salinity levels in seas and oceans due to climate change (Ullah et al. 2021).

To date, only two root-fungus symbioses have been recognised in seagrasses, both involving mycobionts producing relatively thick, melanised hyphae. The first is the endosymbiosis between the dominant Mediterranean seagrass *Posidoniaoceanica* and its specific DS pleosporalean mycobiont, Posidoniomyces (Pos.) atricolor (Vohník et al. 2015, 2019), which appears to be ubiquitous at least in the northwestern Mediterranean Sea. The second example is the epiphytic symbiosis between the Indo-Pacific seagrass Thalassodendron (T.) ciliatum and a DS fungal partner of yet unknown taxonomy, currently documented only from a single site in the northeastern Red Sea (Vohník and Josefiová 2024).

In this study, we focused on describing two previously unknown fungi isolated from distinct, yet understudied, osmotically stressed habitats, namely seagrass roots and saline soils. As part of our investigation into marine symbiotic fungi, we identified multiple isolates of an unknown DS mycobiont exhibiting a unique and previously undescribed colonisation pattern within the roots of *T.ciliatum* from Mauritius. Ten of these strains were included in this study, with five of them successfully sequenced. However, the lack of diagnostic sexual or asexual structures in vivo and in culture prevented their definitive assignment to any established taxon through morphological comparison. The second unidentified fungus is represented by two strains isolated from inland saline soils (Hujslová et al. 2010), an extreme environment where fungal diversity remains insufficiently studied. The sampling site, Soos National Nature Reserve in Czechia, features a variety of soil types affected by salinisation, mineral springs, and bubbling mud volcanoes, known as mofettes, which are associated with dry carbon dioxide emissions. While investigating the diversity of culturable filamentous fungi in this locality, Hujslová et al. (2010) documented 92 fungal taxa. Two strains among these, CCF 3787 and CCF 3788, were identified as *Lulwoana* sp. based on molecular markers (Hujslová et al. 2010). However, no morphological data were recorded at that time. These authors published a BLAST search of the SSU of these strains (GenBank accessions FJ430721 and FJ430723), which revealed 98% identity with *Lulwoanauniseptata* (AY879034, Campbell et al. 2005). The correct name of this species corresponds to its asexual morph, *Zalerionmaritima* (Anastasiou 1963; Nakagiri 1984). *Zalerionmaritima* has so far been recorded exclusively in marine environments, including seawater (Azevedo et al. 2017) and submerged decaying wood and driftwood (e.g. Barghoorn and Linder 1944; Campbell et al. 2005; Gonçalves et al. 2021).

The main objectives of this study were to characterise two unknown fungi and assess their ecological roles and biogeographic distribution. Through integrating phylogenetic analyses with morphological and physiological studies, we aimed to provide a comprehensive assessment of the identity and taxonomic placement of the studied fungal taxa. For this purpose, we generated novel DNA sequences of the internal transcribed spacer (ITS) rDNA along with two other nuclear ribosomal genes for all strains. Using the BLASTn search (Zhang et al. 2000), the ITS sequences of our strains showed similarity to members of the *Lulworthiales*. To clarify their phylogenetic placement within this order, Bayesian Inference and Maximum Likelihood analyses were conducted, incorporating our strains alongside available type and non-type strains of previously described lulworthialean fungi. Furthermore, we examined their morphological variability in culture employing several nutrient media, both with and without seawater, to evaluate their adaptability and morphological responses under differing nutrient and salinity conditions. Incorporation of metabarcoding data in the GlobalFungi database (Větrovský et al. 2020) allowed assessing the distribution and habitat prefer­ences of the fungal species described and drawing broader ecological inferences.

## ﻿Material and methods

### ﻿Sampling

**Figure 1.**
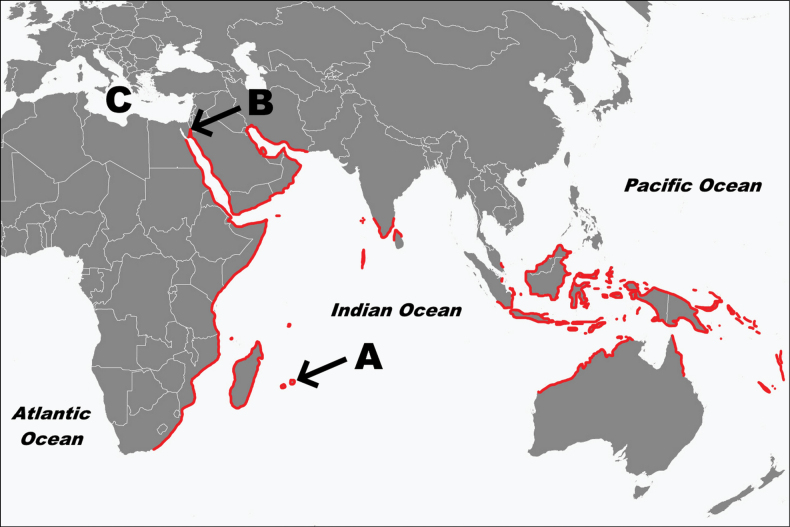
Distribution of *Thalassodendronciliatum* and location of the three known root-fungus symbioses in seagrasses (A–C). A. Location of the current study site on the northeast coast of Mauritius. B. Location of the study site for a novel epiphytic root-fungus symbiosis in *T.ciliatum* in the northeast Red Sea (Vohník and Josefiová 2024). C. The vascular flora of the Mediterranean Sea is dominated by *Posidoniaoceanica*, which forms a specific root-fungus symbiosis with *Posidoniomycesatricolor* (*Pleosporales*) (Vohník et al. 2015, 2019). The distribution of *T.ciliatum* in the Indo-Pacific region (in red) was adapted from Short et al. (2008).

**Figure 2.**
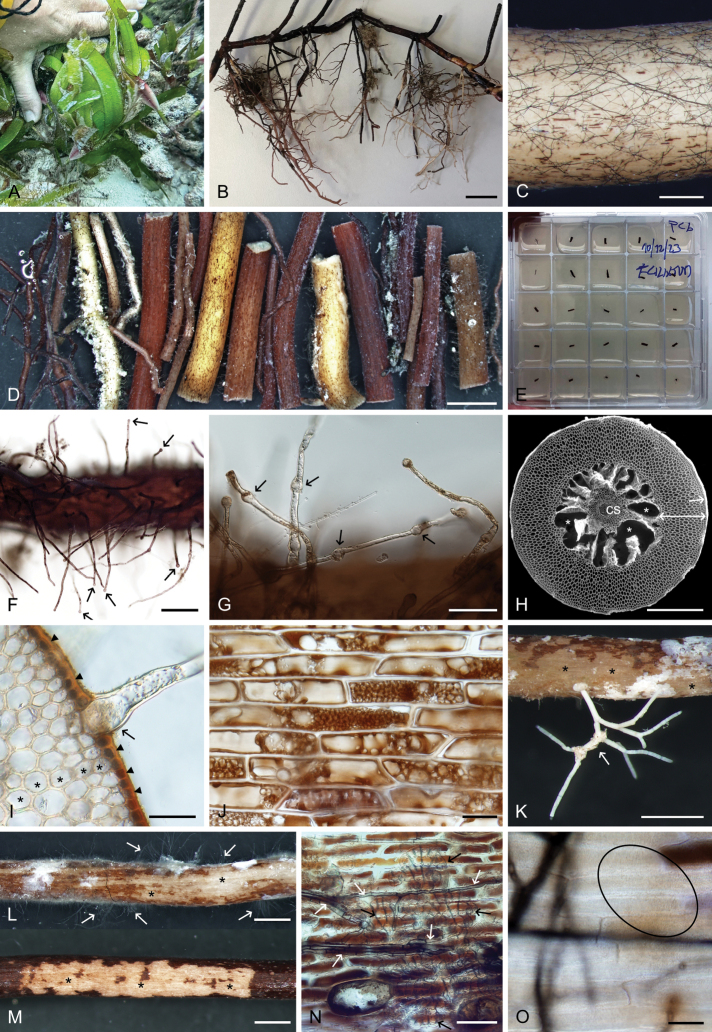
Root traits of *Thalassodendronciliatum* from Mauritius. A. *T.ciliatum* in white coralligenous sand at the sampling site. B. One of the investigated root systems; note the different colours and diameters of individual roots. C. Characteristic fungal colonisation on the surface of a young root; note the thick blackish hyphae and no root hairs (all detached). D. Colour and morphological variability of the screened roots. E. Surface-sterilised root segments plated on a cultivation medium in 25-compartment plastic dishes. F. Root hairs with apical swellings (arrows). G. Intercalated swellings in some root hairs (arrows). H. Transverse section of a typical root showing a single-layered rhizodermis (arrow), a thick cortex (double-headed arrow), an aerenchyma with air lacunae (asterisks), and a central stele (cs). I. Basal part of a hair root (arrow), rhizodermal cells filled with presumed phenolic substances (arrowheads), and significantly larger cortical cells (asterisks). J. Rhizodermal cells variably filled with phenolic substances, sometimes resembling fungal microsclerotia, making differentiation challenging. K–M. Superficial grazing on some roots (asterisk). K, L. Non-fungal epiphytes on the root surface (arrows). N. Complex interplay of fungal (white arrows) and likely bryozoan (black arrows) growth on the root surface. O. Compact, amorphous, translucent layer with a cellular structure at the resolution limits of upright microscopy, possibly of bacterial origin (ellipse), covering many roots. Images: Stereomicroscopy (B–D, F, G, K–M); Scanning electron microscopy (H); Upright microscopy (I, J, N, O). Scale bars: 2 cm (B); 500 µm (C, H); 1 mm (D, K–M); 200 µm (F); 100 µm (G); 50 µm (I, N); 20 µm (J, O).

*Thalassodendronciliatum* (*Alismatales*, *Cymodoceaceae*) was identified based on its characteristic vertical stems, leaf sheaths, and leaf clusters (Green and Short 2003). Sampling was conducted using snorkelling on 7 December 2023 at a site on the northeastern coast of Mauritius (GPS 20°06.98'S, 57°45.17'E) (Fig. 1), where it forms a dense meadow growing in white coralligenous sand (Fig. 2A). Three small, healthy-looking clusters of the seagrass (comprising roots, rhizomes, shoots, and leaves), located approximately 5 m apart, were carefully uprooted by hand at depths of 0.5–1.5 m. Each cluster was placed separately in a plastic bag filled with seawater, stored in a refrigerator, and transported to the laboratory. There, the roots were separated using a scalpel and subjected to microscopic observations and mycobiont isolation, as described below.

### ﻿Microscopic observations of *Thalassodendronciliatum* roots

Stereomicroscopy, light microscopy, and scanning electron microscopy (SEM) were employed to examine the sampled roots for potential fungal colonisation. The procedures followed those described in our previous study on *T.ciliatum* from the Red Sea (Vohník and Josefiová 2024). In brief, all roots were initially screened using an Olympus SZX12 stereomicroscope (Olympus America, Inc., Melville, NY, USA) to detect fungal colonisation on their surface. Handmade longitudinal and transverse sections were prepared from all root types and classified based on their order, diameter, colour, presence or absence of root hairs, and presence or absence of superficial fungal colonisation. These sections were examined under high magnification (400×, 600×, and 1000×) with an Olympus BX60 upright microscope equipped with differential interference contrast (DIC).

Following this qualitative screening, each of the three root samples was divided into two subsamples, one representing the thicker primary roots and the other the thinner secondary roots. From each subsample, 50 randomly selected segments, ca. 0.5 cm in length, were dissected using a razor blade and examined for the presence of superficial DS hyphae and intracellular hyaline hyphae at 200× magnification using the upright microscope (presence/absence data). Data were transformed to percentages of root segments colonised by superficial or intracellular hyphae, and their distribution was assessed for normality. As data on intracellular hyphae violated the assumption of normality, differences in fungal colonisation between primary and secondary roots were assessed by the non-parametric Mann–Whitney U test using the wilcox.test() function in R (R Core Team 2024).

Microphotographs were taken with an Olympus DP70 camera and processed with QuickPHOTO MICRO v. 3.2 software (Promicra, Prague, Czechia). In parallel, samples were examined using an FEI Quanta 200 ESEM scanning electron microscope (FEI Company, Hillsboro, Oregon, USA) in environmental mode at 225 Pa and –12.5°C. Photo documentation was adjusted for clarity and contrast as needed and assembled into figures using Paint.net v. 4.3.12 (dotPDN LLC, Rick Brewster, and contributors).

### ﻿Isolation of *Thalassodendronciliatum* root mycobionts

Many of the screened roots from all three samples displayed a previously undescribed fungal colonisation pattern, which appeared to be formed by a single DS mycobiont. Therefore, rather than characterising all cultivable fungi associated with the sampled roots, our focus was on obtaining pure cultures of the DS mycobiont. To this end, fungi were isolated from the root sample that displayed the highest level of colonisation. The isolation procedure followed methods commonly used in mycorrhizal research, as detailed in Vohník (2020), and routinely applied in our investigations of the mycobiota associated with the roots of the dominant Mediterranean seagrass *P.oceanica* (see Introduction). In brief, healthy-looking colonised roots were identified using a stereomicroscope, separated using a scalpel, surface-sterilised for 60 s in 20% SAVO (common household bleach, Unilever, Prague, Czechia; 100% SAVO contains 47 g/kg^−^¹, i.e. 4.7% sodium hypochlorite = NaClO), washed three times with sterile deionised water, and cut into segments ca. 1–3 mm in length using a flame-sterilised razor blade. Mycobionts were isolated by incubating these segments on the surface of two solid media in plastic square 25-compartment Petri dishes (Fig. 2E): potato carrot agar (PCA; prepared by boiling 40 g of grated carrots and 40 g of grated potatoes separately in 500 mL of deionised water for 5 minutes, the rest of the recipe following Crous et al. 2019), and malt extract agar with mycological peptone (MYP, HiMedia, Mumbai, India; malt extract 30 g, mycological peptone 5 g, and agar 15 g dissolved in 1 L of sterile deionised water). Both media were amended with novobiocin sodium salt (50 mg/L, Sigma-Aldrich, Darmstadt, Germany) to inhibit bacterial growth. After inserting the root segments, the dishes were wrapped with air-permeable tape and incubated in the dark at room temperature. A total of 12 dishes containing 300 root segments were prepared. The isolation process was terminated after 200 days, when no new mycelial growth was observed for 10 consecutive days. Twenty-four randomly selected cultures, two from each dish, were subcultivated on modified Leonian’s agar (MLA) (Malloch 1981) and subsequently transferred to PCA with seawater (PCASW; PCA where deionised water was substituted with seawater).

### ﻿Fungal strains and morphological studies

The strains analysed in this study, along with their sources and newly generated DNA sequences, are detailed in Table 1. All seagrass mycobionts were obtained during this study, while strains isolated from saline continental soils were sourced from the Culture Collection of Fungi (**CCF**), Prague, Czechia. All newly acquired strains are maintained at **CCF** and the Westerdijk Fungal Biodiversity Institute (**CBS**) in Utrecht, the Netherlands. Herbarium specimens (air-dried cultures) were deposited in the Herbarium of the Institute of Botany (**PRA**) in Průhonice, Czechia, and at **CBS**. A representative specimen of *T.ciliatum* was preserved in 50% ethanol and deposited in **PRA** under the accession number PRA-21861.

**Table 1.** Taxa, isolate information, and new sequences determined for this study.

Taxon	Strain	Status	Substrate/host	Country	GenBank Accessions		
					ITS	LSU	SSU
*Halomyrmapluriseptata*	CCF 3788	T	saline soil (pH 7.2)	Czechia	PV441105	PV441119	PV441112
*Halomyrmapluriseptata*	CCF 3787		saline soil (pH 6.8)	Czechia	PV441106	PV441120	PV441113
*Thalassodendromycespurpureus*	CBS 153577	T	root of *Thalassodendronciliatum*	Mauritius	PV441107	PV441121	PV441114
*Thalassodendromycespurpureus*	CBS 153576		root of *Thalassodendronciliatum*	Mauritius	PV441108	PV441122	PV441115
*Thalassodendromycespurpureus*	CBS 153646		root of *Thalassodendronciliatum*	Mauritius	PV441109	PV441123	PV441116
*Thalassodendromycespurpureus*	CBS 153624		root of *Thalassodendronciliatum*	Mauritius	PV441110	PV441124	PV441117
*Thalassodendromycespurpureus*	CBS 153625		root of *Thalassodendronciliatum*	Mauritius	PV441111	PV441125	PV441118

All investigated strains were subjected to in vitro cultivation and morphological characterisation. Mycelium, conidia, and chlamydospore-like structures from living cultures were mounted in water and Melzer’s reagent and examined using an Olympus BX51 compound microscope equipped with DIC. Microscopic structures were photographed with an Olympus DP75 camera operated with Olympus cellSens Dimension software v. 4.3. Colony characteristics were documented using a Canon EOS 77D digital camera fitted with a Canon EF 100 mm f/2.8L Macro IS USM lens (Canon Inc., Tokyo, Japan), illuminated by daylight spectrum 5500K 16W LED lights. Images were processed using Adobe Photoshop CS6 (Adobe Systems, San Jose, USA).

Colony characteristics were assessed on 2-, 4-, and 8-week-old cultures incubated in the dark at 23°C. Several nutrient media were used: cornmeal dextrose agar (CMD; CMA Oxoid Ltd., Hampshire, UK; supplemented with 2% dextrose), MEA (Oxoid), MLA, oatmeal agar (OA), PCA (Crous et al. 2019), PCASW, PDA (Oxoid), and PDA supplemented with NaCl (3.6–3.8%).

### ﻿Gene markers, DNA extraction, PCR amplification, and sequencing

Three molecular markers with the best representation in the *Lulworthiales* were utilised in this study to phylogenetically characterise our strains. The internal transcribed spacer (ITS) rDNA region, comprising ITS1–5.8S–ITS2, serves as the primary barcode for fungi (Schoch et al. 2012) and is valuable for differentiating closely related taxa. Additionally, the nuclear large subunit (LSU) 28S rDNA gene (D1–D3 domains, approximately 1,800 base pairs) and the nuclear small subunit (SSU) 18S rDNA gene (approximately 1,700 base pairs) are widely used markers for investigating relationships within the *Ascomycota*. These markers provide robust phylogenetic resolution at both generic and higher taxonomic levels in fungi (e.g. Spatafora et al. 2006; Schoch et al. 2009).

Genomic DNA was extracted from 8-week-old cultures cultivated on MLA using the DNeasy UltraClean Microbial Kit (Qiagen, Hilden, Germany), following the manufacturer’s instructions for filamentous fungi. The following primers were used to amplify the target genes and gene regions: (1) V9G/LR8 primer pair (de Hoog and Gerrits van den Ende 1998; Vilgalys unpubl.) for the ITS–LSU region and (2) NSSU131/NS24 (Gargas and Taylor 1992; Kauff and Lutzoni 2002) for the SSU region. PCR amplifications of ITS, LSU, and SSU using the Q5 High Fidelity DNA Polymerase Kit (New England Biolabs Inc., Hitchin, UK) were carried out as described in Réblová et al. (2020). Each amplicon was sequenced in both directions using both the PCR and nested primers: ITS5, ITS4, JS1, JS7, JS8, and LR6 for the ITS–LSU region (Vilgalys and Hester 1990; White et al. 1990; Landvik 1996; Vilgalys unpubl.), and NSSU1088, NSSU897R, SR7R, and NS6 for SSU (White et al. 1990; Kauff and Lutzoni 2002; Vilgalys unpubl.). Sequencing was performed by Eurofins Biotech Sequencing Service (Cologne, Germany). The raw sequence data were assembled, reviewed, and edited using Sequencher v. 5.4.6 (Gene Codes Corp., Ann Arbor, USA).

### ﻿Phylogenetic analyses

As a result of BLAST searches, optimised for the blastn and megablast algorithms, of ITS, LSU, and SSU sequences of our strains, available sequences from representatives of the *Lulworthiales* and the related order *Koralionastetales (Lulworthiomycetidae)* were retrieved from the GenBank sequence database at NCBI (Sayers et al. 2019) and incorporated into the analyses. The accession numbers of the obtained sequences, along with their references, are provided in Table 2.

**Table 2.** Taxa, isolate information, and sequences retrieved from GenBank.

Taxon	Strain	Status	Substrate/host	Country	GenBank Accessions			Reference
					ITS	LSU	SSU	
*Achroceratosphaeriapotamia*	CBS 125414	T	submerged wood of *Platanus* sp.	France	MH863679	GQ996538	GQ996541	Réblová et al. 2010
*Cumulosporamarina*	MF46		submerged wood	Egypt	–	GU252135	GU252136	Abdel-Wahab et al. 2010
*Halazoonfuscus*	NBRC 105256		driftwood	Japan	–	GU252147	GU252148	Abdel-Wahab et al. 2010
*Halazoonmelhae*	MF819	T	drift stems of *Phragmitesaustralis*	Egypt	–	GU252143	GU252144	Abdel-Wahab et al. 2010
Halophilomyceshongkongensis*	HOMAR2	T	rhizomes of *Halophilaovalis*	China	PP350735	PP347859	PP347845	Wang et al. 2024
*Hydeapygmea*	NBRC 33069		driftwood	Japan	–	GU252133	GU252134	Abdel-Wahab et al. 2010
*Kohlmeyeriellacrassa*	NBRC 32133	T	sea foam	Japan	LC146741 ^a^	LC146742 ^a^	AY879005	Campbell et al. 2005; ^a^Unpublished
*Kohlmeyeriellatubulata*	PP 1105		marine environment	Denmark	–	AF491265 ^b^	AY878998	Campbell et al. 2002^b^, 2005
*Koralionastesellipticus*	J.K. 5769		on coral rocks with sponges	Belize	–	EU863585	EU863581	Campbell et al. 2009
*Lindracrassa*	ATCC 56663	T	thalli of *Sargassum* sp.	Belize	–	–	AY878999	Campbell et al. 2005
*Lindraobtusa*	NBRC 31317	T	sea foam	Japan	LC146744 ^a^	AY878960	AY879002	Campbell et al. 2005; ^a^Unpublished
*Lindrathalassiae*	J.K. 5090A		detritus, marine environment	USA	DQ491508	DQ470947	DQ470994	Schoch et al. 2009
*Lulwoidealignoarenaria*	NBRC 32135		sea foam	Japan	–	AY878968	AY879010	Campbell et al. 2005
*Lulworthiaatlantica*	FCUL090707CF10	T	sea water	Portugal	KT347213	JN886814	KT347199	Azevedo et al. 2017
*Lulworthiafucicola*	ATCC 64288	N	intertidal wood	Chile	–	AY878965	AY879007	Campbell et al. 2005
*Lulworthiamedusa*	J.K. 5581		culm of *Spartinaalterniflora*	USA	–	AF195637	AF195636	Kohlmeyer et al. 2000
*Lulworthiaopaca*	CBS 218.60		driftwood in seawater	USA	–	AY878961	AY879003	Campbell et al. 2005
Lulworthiacf.purpurea	FCUL170907CP5		sea water	Portugal	KT347219	JN886824	KT347201	Azevedo et al. 2017
*Lulworthiaceae* sp.	BN1 clone4G		coral reef	Caroline Island	–	KU359242	–	Unpublished
*Lulworthiaceae* sp.	BN1 clone4J		coral reef	Caroline Island	–	KU359244	–	Unpublished
*Matsusporiumtropicale*	NBRC 32499		submerged wood of *Rhizophorastylosa*	Japan	–	GU252141	GU252142	Abdel-Wahab et al. 2010
*Moleosporamaritima*	MF 836	T	drift stems of *Phragmitesaustralis*	Egypt	–	GU252137	GU252138	Abdel-Wahab et al. 2010
*Moromycesvarius*	GR78		submerged wood	Thailand	–	EU848578	EU848593	Jones et al. 2008
*Orbimycesspectabilis*	n/a		n/a	n/a	–	–	EU848580	Jones et al. 2008
*Paralulworthiacandida*	MUT 5430	T	roots of *Posidoniaoceanica*	Italy	MZ357724	MZ357746	MZ357767	Poli et al. 2021
*Paralulworthiaelbensis*	MUT 5422	T	roots of *Posidoniaoceanica*	Italy	MZ357723	MZ357745	MZ357766	Poli et al. 2021
*Paralulworthiagigaspora*	MUT 435	T	rhizomes of *Posidoniaoceanica*	Italy	MN649242	MN649250	MN649246	Poli et al. 2020
*Paralulworthiahalima*	CBS 208.64	T	submerged wood of *Tamarixaphylla*	USA	MH858421	MH870049	—	Vu et al. 2019
*Paralulworthiahalima*	MUT 3347		submerged wood	Italy	MZ357728	MZ357750	MZ357771	Poli et al. 2021
*Paralulworthiamediterranea*	MUT 5417	T	roots of *Posidoniaoceanica*	Italy	MZ357721	MZ357743	MZ357764	Poli et al. 2021
*Paralulworthiaposidoniae*	MUT 5261	T	rhizomes of *Posidoniaoceanica*	Italy	MN649245	MN649253	MN649249	Poli et al. 2020
*Paramoleosporaguttulata*	CGMCC 3.22494	T	*sediment*	China	OQ798961	OQ758156	OQ758189	Li et al. 2023
*Pisorisporiumcymbiforme*	CBS 138884	T	submerged wood of *Alnusglutinosa*	France	–	KM588904	KM588901	Réblová et al. 2015
*Pontogeneiamicrodictyi*	J.K. 5748	P	thalli of *Microdictyon* sp.	Bahamas	–	–	EU863582	Campbell et al. 2009
*Rambelliseagigliensis*	HPa3	T	tunic of *Halocynthiapapillosa*	Italy	OR367423	OR369726	OR371466	Braconcini et al. 2024
*Rambelliseahalocynthiae*	HPa50		tunic/internal tissues of *Halocynthiapapillosa*	Italy	OR367481	OR371457	OR371485	Braconcini et al. 2024
*Rostrupielladanica*	BBH 16759	T	submerged wood of *Fagussylvatica*	Denmark	–	DQ394094	–	Koch et al. 2007
*Sammeyersiagrandispora*	J.K. 4686		dead branch of *Rhizophora* sp.	Belize	–	DQ522856	DQ522855	Schoch et al. 2009
*Spathulosporaadelpha*	J.K. 5599		thalli of *Balliacallitricha*	Australia	AY380314	AY380314	AY380314	Inderbitzin et al. 2004
*Spathulosporaantarctica*	J.K. 3530		thalli of *Balliacallitricha*	Argentina	AY380315	AY380315	AY380315	Inderbitzin et al. 2004
*Zalerionmaritima*	FCUL280207CP1		sea water	Portugal	KT347216	JN886806	KT347203	Azevedo et al. 2017
*Zalerionmaritima*	NBRC 32137^#^	T	submerged wood	Japan	LC146746 ^a^	LC146746 ^a^	AY879031	Campbell et al. 2005; ^a^Unpublished
*Zalerionpseudomaritima*	CMG 65	T	submerged wood	Portugal	MT235733	MT235750	MT235709	Gonçalves et al. 2021

Notes: T, N, and P denote ex-type, ex-neotype, and ex-paratype strains. * Nom. inval. Art. 40.7. # ex-holotype of *Lulworthiauniseptata*. n/a = data not available. submerged wood and driftwood refer to the substrate submerged in seawater.

All sequences were aligned using MAFFT v. 7.487 (Katoh and Standley 2013), implemented in the CIPRES Science Gateway v. 3.3 (Miller et al. 2010), and manually corrected in BioEdit v. 7.1.8 (Hall 1999) when necessary. Bayesian Inference (BI) and Maximum Likelihood (ML) analyses were conducted to evaluate the phylogenetic relationships of our isolates, using software packages available through the CIPRES Science Gateway v. 3.3. The ML analysis was performed using RAxML-HPC v. 8.2.12 with a GTRCAT approximation (Stamatakis 2014). Node support was assessed through non-parametric bootstrapping (BS) with 1,000 replicates. The BI analysis was conducted using MrBayes v. 3.2.7 (Ronquist et al. 2012), with two independent Bayesian searches run under default parameters. The Bayesian Metropolis-coupled Markov chain Monte Carlo (B-MCMCMC) analyses continued until the average standard deviation of split frequencies fell below 0.01. Trees were sampled every 1,000 generations, with a burn-in of 25%. The BI and ML phylogenetic trees were visually compared to assess topological conflicts among supported clades. Phylogenetic trees were visualised using FigTree v. 1.4.3 (Rambaut 2010) and SeaView v. 5.0.5 (Gouy et al. 2010) and further edited in Microsoft PowerPoint and CorelDRAW Graphics Suite v. 25.2.1.313 (Alludo, Ottawa, Canada).

Separate ML analyses of single-marker sequence alignments were performed. As no conflicting clades were identified in these analyses, the individual alignments of ITS, LSU, and SSU sequences were combined into a concatenated multi-locus alignment, which was subsequently used for phylogenetic analyses. The alignment is available as Suppl. material 1. The best-fit models of nucleotide evolution for each partition (ITS, LSU, SSU), under Akaike information criterion, were determined using jModelTest v. 2.1.10 (Guindon and Gascuel 2003; Darriba et al. 2012): GTR+G for ITS, TIM1+I+G for LSU, and TIM2+I+G for SSU.

The alignment encompassed 49 ingroup strains and consisted of a total of 4,366 characters, including gap regions, with 1,631 unique character sites identified by RAxML: 457 in ITS, 623 in LSU, and 551 in SSU. Specific regions were excluded due to incomplete sequences in GenBank, including 116 nucleotides (nt) of LSU and 99 nt of SSU at the 5′ end, as well as 509 nt of LSU at the 3′ end. Two members of the order *Pisorisporiales*, namely *Achroceratosphaeriapotamia* and *Pisorisporiumcymbiforme*, were chosen as the outgroup based on prior studies and established relationships of lulworthialean taxa within the *Sordariomycetes* (Réblová et al. 2015).

The AI-generated figure in the background of the phylogenetic tree was created with ChatGPT (OpenAI 2025, version Feb 2025, retrieved on 10 February 2025).

### ﻿Environmental sequence data as a tool for biogeographical assessment

The geographic distribution of the two newly described species was evaluated through published environmental ITS sequences using the methodology described by Réblová et al. (2022). For this analysis, we accessed the GlobalFungi database (Větrovský et al. 2020), release 5 (16 November 2023), which includes 84,972 samples from 846 studies and comprises a total of 593,399,355 ITS sequence variants. Since GlobalFungi stores ITS1 and ITS2 sequences separately, we processed these regions independently. To ensure alignment with the ITS spacers archived in GlobalFungi, we extracted the relevant spacers from our dataset using the ITSx extractor within the SEED2 platform (Větrovský et al. 2018). Identification of our species in GlobalFungi was performed through an exact match similarity search, comparing all unique ITS1 and ITS2 haplotypes from our study against published environmental sequences that matched in both length and nucleotide composition.

## ﻿Results

### ﻿Microscopic observations of *Thalassodendronciliatum* roots

**Figure 3.**
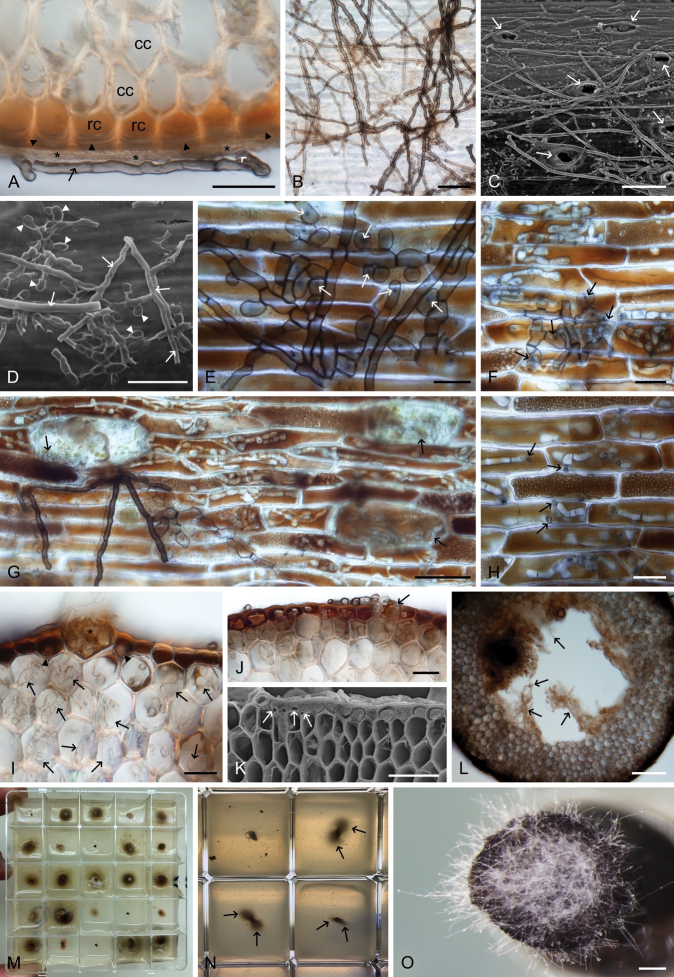
Fungal colonisation on and within the roots of the seagrass *Thalassodendronciliatum*. A. Transverse section showing cortical cells (cc), rhizodermal cells (rc) filled with phenolic compounds and exhibiting thickened outer cell walls (arrowheads), an unidentified substance on the root surface (asterisks), and a melanised septate hypha (arrow). B. Superficial fungal colonisation showing various degrees of hyphal melanisation. C. Superficial fungal colonisation; note the root hair scars (arrows). D. Two types of surface hyphae: longer rounded (arrows) and shorter flattened (arrowheads). E. Flattened hyphae resembling appressoria with thin penetration hyphae (arrows). F. Gradual transition from superficial fungal colonisation with melanised hyphae to intracellular colonisation with hyaline hyphae, facilitated by penetration hyphae (arrows). G. Key features of the novel root-fungus symbiosis: two types of superficial hyphae (filamentous and flattened with penetration hyphae), intracellular hyaline hyphae within the rhizodermis, and colonisation within the root hair scars (arrows). H. Penetration hyphae (arrows). I. Fungal colonisation within a root hair scar (asterisk), the rhizodermis (arrowheads), and the cortex (arrows). J. Fungal colonisation on the root surface, within a root hair scar (arrow), and in the rhizodermis and the cortex. K. Hyphae on the root surface and within the rhizodermis (arrows). L. Fungal hyphae reaching the stele (arrows). M. Root segments with emerging fungal colonies after 200 days of incubation. N. Mycelia emerging from the inner part of an incubated root segment (arrows). O. Mycelium emerging from the inner part of an incubated root segment. Images: Upright microscopy (A, B, E–J, L); stereomicroscopy (O); scanning electron microscopy (C, D, K). Scale bars: 20 µm (A, E, F, H–J); 50 µm (B, D, G, K); 100 µm (C, L); 200 µm (O).

Although a few roots appeared moribund, the majority were turgid and appeared healthy (Fig. 2B–D). Their colour ranged from whitish to dark brown to black, and their diameter from 561 ± 84 µm (mean ± SD; min. 376, max. 711, n = 25) for primary roots to 152 ± 65 µm (min. 89, max. 343, n = 25) for secondary roots (Fig. 2D). Even under low magnification (stereomicroscope), many roots showed superficial colonisation by relatively thick, dark brown to black fungal hyphae (Fig. 2C). Most roots exhibited root hairs, their remnants (oval-shaped basal parts), or pit-like depressions left after their complete detachment (root hair scars, Figs 2D, 3C). Similar to *T.ciliatum* from the Red Sea (Vohník and Josefiová 2024), some root hairs displayed apical swellings that likely aid in seagrass anchoring within the seabed substrate (Fig. 2F), resembling those occurring in *P.oceanica* (Kolátková and Vohník 2019). Additionally, some root hairs with apical swellings possessed intercalated swellings—often multiple—morphologically resembling the “circular swelling zones” reported in *P.oceanica* by Tomasello et al. (2018) (Fig. 2G).

Transverse sections revealed a rhizodermis composed of relatively small, flattened cells, a thick cortex, an aerenchyma with air lacunae, and a central stele (Fig. 2H, I). In older roots, most rhizodermal cells were filled with what is here interpreted as phenolic compounds (Fig. 2J), resembling the “tannin cells” previously reported in *T.ciliatum* from the Red Sea (Vohník and Josefiová 2024). Exposed roots, meaning those occurring above the seabed substrate, were frequently colonised by various micro- and macroscopic non-fungal epiphytes (Fig. 2K, L), some of them resembling structures formed by fungi (Fig. 2N), namely hyphal mantles or pseudoparenchymatous nets on the root surfaces of certain seagrasses (Vohník et al. 2015; Vohník and Josefiová 2024), and “mycélium en palmettes” formed on leaf cuticles (Le Renard et al. 2021). Many exposed roots displayed signs of grazing (Fig. 2K–M). Similarly to *T.ciliatum* from the Red Sea, many roots were covered by a compact, amorphous, translucent layer with a cellular structure at the resolution limits of light microscopy (Fig. 2O). This layer did not have any apparent negative effect on the development of fungal hyphae, which often grew on its surface (Fig. 3A), and was morphologically identical to that observed in *T.ciliatum* from the Red Sea (see fig. 4G–K in Vohník and Josefiová 2024). Fungal colonisation was observed on both thick primary and thin secondary roots, with no apparent correlation to the presence of root hairs (cf. Borovec and Vohník 2018; Vohník and Josefiová 2024). The surface hyphae ranged in colour from hyaline to light brown when young, becoming dark brown with age. They were either straight or undulating (Fig. 3B, C) but did not form the characteristic patterns observed in the symbioses in *P.oceanica* and *T.ciliatum* from the Mediterranean and Red Sea, respectively. One distinctive feature of this colonisation was the presence of flattened and shortened club-like hyphae resembling appressoria, which indeed seemed to relate to intracellular penetration of the host rhizodermis via thin penetration hyphae (Fig. 3D–H). Another unique aspect of this novel symbiosis was the intracellular colonisation of host rhizodermal cells filled with phenolic compounds by hyaline, rounded, septate hyphae of varying diameter and relatively thin cell walls (Fig. 3F–H). Cell-to-cell colonisation was observed in both longitudinal and transverse sections, with thin hyphae penetrating host cell walls (Fig. 3F–J). Root hair scars were often filled with fungal mycelia (Fig. 3G, I, J), though it remained unclear whether these scars functioned as entry or exit points during the colonisation process. The intraradical fungal colonisation extended from the surface hyphae through the rhizodermis and cortex, spreading via the septa of the aerenchyma toward the stele (Fig. 3I–L).

There were no statistically significant differences in the incidence of superficial (U = 3.5, p = 0.825) or intracellular (U = 5.0, p = 1.0) hyphae between primary and secondary roots. The incidence of superficial hyphae was 53 ± 31% (mean ± SD) and 58 ± 27%, while the incidence of intracellular hyphae was 15 ± 18% and 14 ± 21% for primary and secondary roots, respectively.

### ﻿Isolation of *Thalassodendronciliatum* root mycobionts

Fungal growth was observed exclusively on PCA, with no fast-growing or sporulating fungi detected. All emerging colonies exhibited uniform morphology and colouration, characterised by relatively slow-growing whitish mycelium that gradually transitioned from ochre to a light brown shade (Fig. 3M). Initial hyphal growth from the root segments was observed after ca. 2 weeks, often emerging from the open ends of the root segments, i.e. from the inner root tissues (Fig. 3N, O). By the end of the isolation process, which lasted 200 days, a total of 119 morphologically uniform fungal isolates were obtained from the 300 surface-sterilised root segments, resulting in an overall success rate of 39.7%. However, there were apparent differences between the dishes, with one yielding 24 isolates (96%) and three displaying no fungal growth (0%). Five random segments were picked from each of these three dishes and checked for fungal colonisation using light microscopy as described above. All possessed DS hyphae on their surface, confirming the effectiveness of the root surface sterilisation as well as the visually observed intraradical origin of the obtained isolates.

### ﻿Phylogenetic analyses

**Figure 4.**
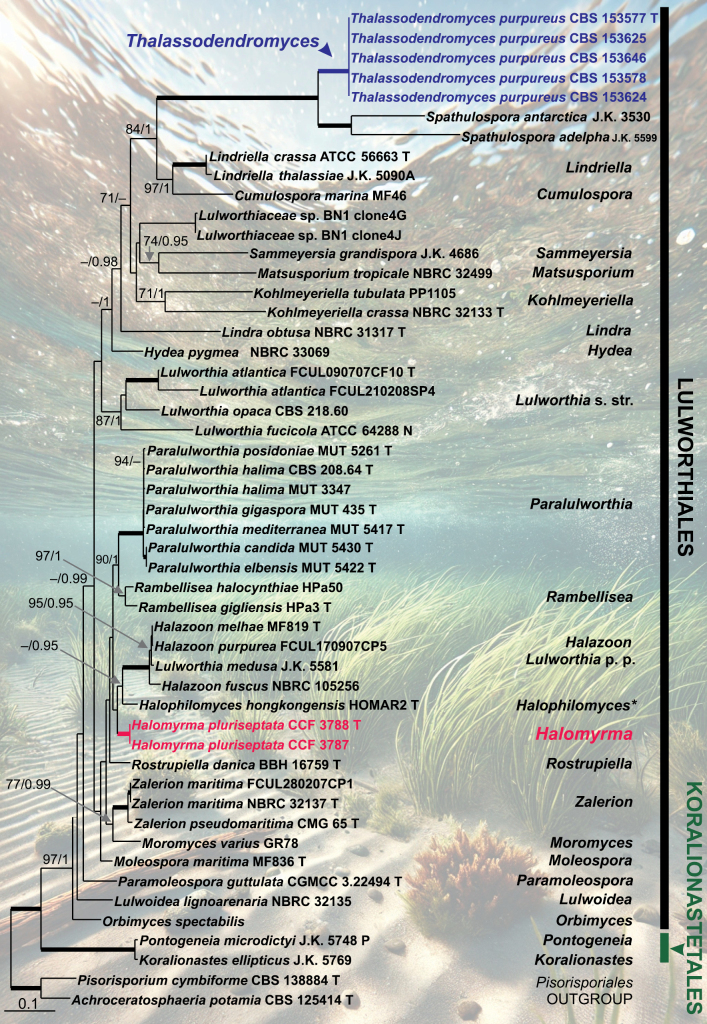
Maximum likelihood phylogenetic tree of members of the *Lulworthiomycetidae* based on the concatenated matrix of ITS, LSU, and SSU sequences. Names highlighted in red and blue indicate taxonomic novelties. T, N, and P denote ex-type, ex-neotype, and ex-paratype strains, respectively. An asterisk (*) indicates Nom. inval. (ICN, Art. 40.7). Thickened branches indicate support MLBS = 100% and PP values = 1.0. Branch support of nodes ≥ 70% ML and ≥ 0.95 PP is indicated above or below branches.

Phylogenetic analyses of the ITS, LSU, and SSU sequences were conducted to assess the relationships of our isolates with members of the *Lulworthiales* and the closely related *Koralionastetales*. The topologies of phylogenetic trees constructed using BI and ML methods were largely consistent. Nodes with support values of ≥70% ML bootstrap (BS) and ≥0.95 Bayesian posterior probability (PP) were regarded as well supported. The ML phylogenetic tree is presented in Fig. 4. The subclass *Lulworthiomycetidae* (100% MLBS/1 PP) comprises two strongly supported clades: *Koralionastetales* (100/1) and *Lulworthiales* (97/1). Within the *Lulworthiales*, 23 distinct lineages have been identified, corresponding to genera and morphologically defined species groups. The recovered lineages are consistent with those described by Dayarathne et al. (2025). *Lulworthia* was found to be paraphyletic, with the *Lulworthia* s. str. clade (87/1) and an additional species, *L.medusa*, positioned within the *Halazoon* clade (100/1). The genus *Lindra* is represented by the ex-type strain of *Li.obtusa* (NBRC 31317), whereas the two species with available DNA data have been transferred to the newly established genus *Lindriella* (Dayarathne et al. 2025). However, due to the absence of molecular data for *Li.inflata*, the type species, it is not possible to determine whether this clade represents the core of the genus.

*Haloguignardiairritans*, currently the only known representative of the genus with an available DNA sequence, was not included in the final phylogenetic analyses. The available molecular data belong to a strain isolated from the thalli of *Cystoseiraosmundacea* in the USA (with no cited strain or herbarium material) and are limited to a partial SSU sequence (AY566252), with a reference to Harvey et al. (2010); however, the sequence was not included in the study. In preliminary analysis (results not shown), *H.irritans* formed a long branch with an unresolved position in the *Paralulworthia* clade, but its phylogenetic affiliation remains uncertain. Additionally, molecular data for *Haloguignardiadecidua* (Cribb and Cribb 1956), the type species of the genus, are unavailable, preventing a definitive placement of *Haloguignardia* within the *Ascomycota*.

Two strains, CCF 3787 and CCF 3788, isolated from saline inland soils, formed a strongly supported monophyletic clade (100/1). This clade was recovered as a sister group to *Halazoon* and *Halophilomyces*, although this relationship lacked statistical support. These strains are introduced as a new genus and species, *Halomyrmapluriseptata* gen. et sp. nov. Five isolates—CBS 153624, CBS 153625, CBS 153646, CBS 153577, and CBS 153578—representing the seagrass root-associated mycobiont, formed a strongly supported clade (100/1) positioned as a sister to *Spathulospora*, which was represented by *S.adelpha* J.K. 5599 and *S.antarctica* J.K. 3530. These isolates are designated as the new genus and species, *Thalassodendromycespurpureus* gen. et sp. nov. A well-supported lineage (97/1), comprising *Lindriella* and *Cumulospora*, was resolved as the closest relative (84/1) of the *Spathulospora*–*Thalassodendromyces* clade.

### ﻿Biogeographic analysis

The search in the GlobalFungi database for identical ITS sequences to *T.purpureus* did not return any results, suggesting that this species is a specialised mycobiont of the seagrass *T.ciliatum*. In contrast, 71 samples with identical sequences were retrieved for *H.pluriseptata*. Metadata for both ITS1 (59 samples) and ITS2 (12 samples) sequences indicate association with aquatic environments (86%), particularly marine, estuarine, and occasionally freshwater ecosystems, where it was detected in sediment, soil, shoots, and plant roots (*Zosteramarina*, *Potamogeton* sp.). The presence of *H.pluriseptata* in wetlands and coastal dune soils further adds evidence to its affinity for moisture-rich habitats. Additional occurrences were recorded in terrestrial environments (14%), including grasslands, croplands, shrublands, wetlands, and anthropogenic biomes. Overall, the occurrences across biomes were most frequently associated with sediment (69%), followed by soil (16%), plant roots (13%), and shoots (2%). Primary occurrence data and associated metadata, including study ID in GlobalFungi, location, sample type, biome, pH, and climatic variables such as mean annual precipitation (MAP) and mean annual temperature (MAT), were retrieved from the GlobalFungi database and are available as Suppl. material 2.

### ﻿Taxonomy

#### ﻿ Halomyrma

Taxon classificationAnimaliaLulworthialesLulworthiaceae

Réblová & Vohník gen. nov.

A4CBD1E1-572C-536B-85A2-41D40908D8EC

MB857649

##### Etymology.

From Greek *háls* (salt or saline) and *mýrmēx* (ant or metaphorically ‘small, resilient organism’) Referring to the habitat of the fungus in saline soil and symbolising its adaptability and survival in extreme environments.

##### Type species.

*Halomyrmapluriseptata* Réblová & Vohník.

##### Description.

**Asexual morph.** Observed exclusively in culture. **Mycelium** composed of cylindrical, subhyaline to olivaceous brown, septate, branched hyphae. **Conidiophores** micronematous, reduced to undifferentiated hyphal branches or single conidiogenous cells. **Conidiogenous cells** holoblastic, cylindrical, subcylindrical, or slightly swollen, determined; conidial secession schizolytic. **Conidia** dry, terminal, solitary, initially coiled and composed of several cells, gradually becoming ellipsoidal, subglobose, clavate, or irregular in shape, multicellular, with transverse and longitudinal septa; cells are formed in multiple planes, pigmented, with a pore in each cell. **Sexual morph.** Not observed.

#### ﻿ Halomyrma pluriseptata

Taxon classificationAnimaliaLulworthialesLulworthiaceae

Réblová & Vohník sp. nov.

DD5A3811-BEC3-5E21-8330-5CBA468F6DF6

MB857651

Figs 5

##### Etymology.

From Latin *pluri* (many or multiple) and *septatus* (partitioned or divided by septa). Referring to the characteristic multiseptate conidia.

##### Type.

CZECHIA • Karlovy Vary Region, Cheb district, Soos National Nature Reserve near Františkovy Lázně; slightly saline soil (pH 7.2); 2005; leg. & isol. M. Hujslová M.H. 779 (**holotype**PRA-22368 dried culture on CMD, ex-type culture CCF 3788 = CBS 153583, **paratype**PRA-22369 dried culture on MLA). GenBank: ITS = PV441105, LSU = PV441119, SSU = PV441112 (this study); SSU, ITS, LSU = FJ430723 (Hujslová et al. 2010).

##### Culture characteristics.

On CMD colonies 50–55 mm diam. in 2 wk, circular, slightly raised, margin flat and entire, whitish centrally, grey towards the margin due to formation of immersed conidia, floccose at the inoculation block, cobwebby towards the periphery, reverse dark olivaceous grey. On MLA colonies 35–36 mm diam. in 2 wk, convex, margin flat and entire, irregularly whitish-grey and floccose at the centre, grey and cobwebby towards the periphery, olivaceous grey at the margin, reverse olivaceous grey. On OA colonies 39–43 mm diam. in 2 wk, circular, flat, margin entire, floccose and partially funiculose at the inoculation block, cobwebby to mucoid towards the periphery, white at the centre, irregularly brown towards the margin, a deep golden-yellow pigment diffusing into the agar, reverse golden-yellow with dark olivaceous brown spots. On PCA colonies 33–40 mm diam. in 2 wk, convex, margin flat and entire, floccose, white at the centre, irregularly dark grey towards the periphery due to abundant immersed conidia, a pale yellow pigment diffusing into the agar, reverse yellow or dark olivaceous brown. On PCASW colonies 28–30 mm diam. in 2 wk, circular, flat, margin entire, lanose, sparse towards the periphery, whitish to creamy centrally, mouse grey at the margin, with conspicuous submerged growth, yellow pigment diffusing into the agar, reverse creamy or yellow. Sporulation abundant on all media, conidia formed on aerial as well as vegetative hyphae immersed in the agar.

**Figure 5.**
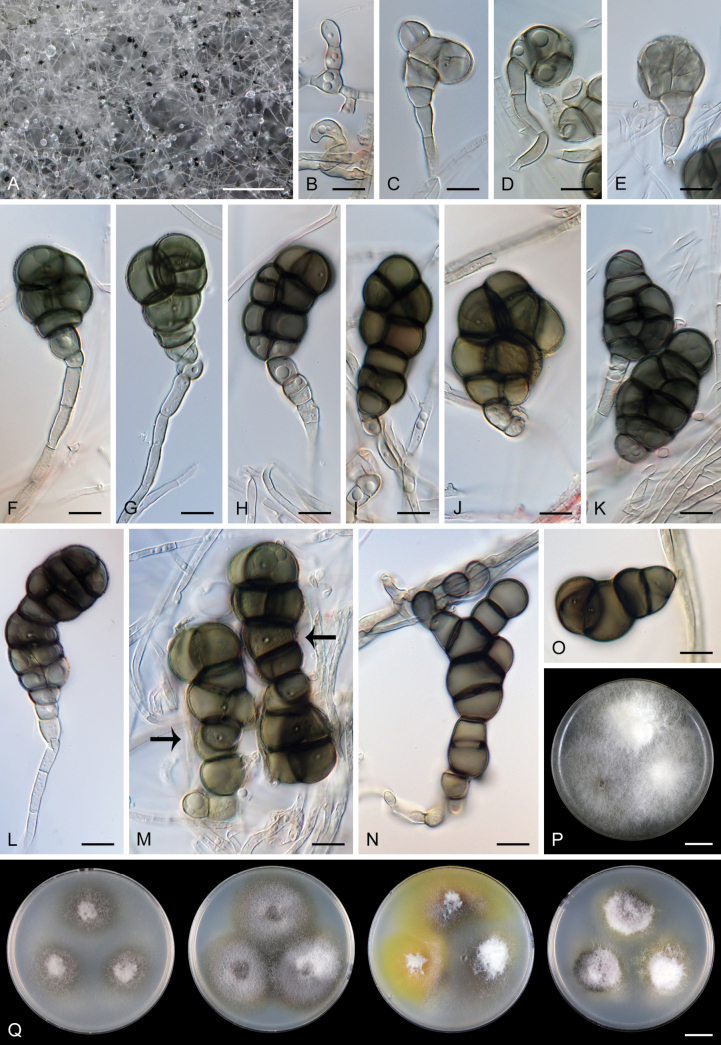
*Halomyrmapluriseptata* (ex-type strain CCF 3788). A. Colony; conidia growing on aerial hyphae. B–E. Early stages of conidial development. F–O. Fully developed conidia, exhibiting variability in shape (arrows indicate pores in the walls). P. Colony morphology on PCASW after 3 wk. Q. Diversity of colony morphology on CMD, MLA, OA, and PCA, respectively (from left to right) after 2 wk. Images: on MEA (A, I, M–O); on PCA (B–H, J–L). Scale bars: 500 µm (A); 10 µm (B–O); 1 cm (P, Q).

##### Description in culture.

**Asexual morph.** On PCA, colonies effuse, **mycelium** composed of hyphae 1.5–3.5(–4) μm wide, cylindrical, subhyaline to olivaceous brown, septate, branched. **Conidiophores** micronematous, reduced to undifferentiated hyphal branches or single conidiogenous cells. **Conidiogenous cells** 4.5–12 × 3.5–6.5(–8.5) μm, holoblastic, conidial secession schizolytic, terminal or intercalary, inconspicuous, subhyaline to olivaceous brown, determined, smooth. **Conidia** dry, terminal, solitary, of various shapes: cylindrical with usually transverse septa 55–85(–104) × 14–19(–25) μm (mean ± SD = 75.6 ± 15.3 × 18.2 ± 3.6 μm), mostly ellipsoidal, subglobose, clavate, or irregular in shape and multicellular (dictyoconidia) 31.5–63.5(–77) × 20.5–27.5(–37) μm (mean ± SD = 50.2 ± 11.7 × 22.8 ± 2.4 μm), brown to dark olivaceous brown, smooth, but some conidia appeared finely rugose, with a pore 1–1.5 μm diam. in each cell, slightly constricted at the septa, dark brown, darker at the septa, basal cell clavate or somewhat ellipsoidal, subhyaline to pale olivaceous brown. Initially, conidia possess only transverse septa and are slightly coiled; upon maturation, longitudinal septa may develop, as well as additional cells formed in multiple planes, giving rise to more compact structures. **Sexual morph.** Not observed.

##### Additional specimen examined.

CZECHIA • Karlovy Vary Region, Cheb district, Soos National Nature Reserve near Františkovy Lázně; slightly saline soil (pH 6.8); 2005; leg. & isol. M. Hujslová M.H. 158 (paratype PRA-22497 dried culture on MLA, culture CCF 3787 = CBS 153584).

##### Habitat and geographical distribution.

*Halomyrmapluriseptata* was isolated from slightly saline inland soils (pH 6.8–7.2) in Czechia. According to GlobalFungi, *H.pluriseptata* has a broad geographical distribution, spanning from tropical to temperate climatic zones. Identical ITS sequences were found in 71 samples from aquatic and terrestrial habitats. Among aquatic biomes, *H.pluriseptata* was identified in marine salt marsh sediments in the UK (Alzarhani et al. 2019) and Massachusetts, USA (Lynum et al. 2020), as well as in marine sediments and roots of the seagrass *Zosteramarina* in Croatia and Alaska, USA (Ettinger et al. 2021). Additional marine records include occurrences in the neritic benthic zone in Sweden (Retter et al. 2019). In freshwater environments, the fungus was found in lake sediments in China (Zhang et al. 2021) and in *Potamogeton* sp. roots in California, USA (Ettinger et al. 2021). A notable occurrence was also recorded in the rhizosphere soil in a hypersaline lake in China (Luo et al. 2021), suggesting its adaptability to high-salinity freshwater systems. In contrast, *H.pluriseptata* was less frequently recorded in terrestrial biomes. It was identified in grassland soils in the UK (George et al. 2019) and Denmark (Frøslev et al. 2019), as well as coastal dune soils within wetland ecosystems in Canada (Roy-Bolduc et al. 2015). Further occurrences include sediments from a shrubland biome in California, USA (Lin et al. 2021), and soil samples from cropland fields in China (Zhao et al. 2018) and the Netherlands (van Rijssel et al. 2022). In other anthropogenic environments, the fungus was detected in plantation rhizosphere soil in China (Huang et al. 2020) and topsoil in Poland (Okrasińska et al. 2022), indicating its ability to persist in disturbed habitats.

##### Notes.

Hujslová et al. (2010) documented four strains of *Lulwoana* sp. (CCF 3787, CCF 3788, M.H. 585, and M.H. 630) from saline soils. However, a BLASTn search of their published sequences indicated that strain M.H. 585 (GenBank accession FJ430720) is affiliated with the *Magnaporthaceae*, while the other three isolates have affinity with the *Lulworthiales*. Consequently, two strains, CCF 3787 and CCF 3788, were included in our study and are now recognised as *H.pluriseptata*, while strain M.H. 630 was unavailable for examination.

The cultural characteristics and description of *H.pluriseptata* are based on the ex-type strain CCF 3788. While both strains shared identical ITS, LSU, and SSU rDNA sequences and exhibited comparable conidial morphology, they displayed differences in cultural characteristics. The non-type strain CCF 3787 demonstrated slightly faster growth on MLA, OA, and PCA (i.e. 50–52 mm in diam. on CMD, 44–46 mm on MLA, 62–64 mm on OA, 48–55 mm on PCA, and 24–25 mm on PCASW) within the same incubation period. Additionally, the ex-type strain CCF 3788 produced pigments that diffused into the agar: golden-yellow on OA and yellow on PCA and PCASW, whereas CCF 3787 did not exhibit pigment production on any tested medium.

Additionally, PDA and PDA supplemented with NaCl were used to further assess the growth and salt tolerance of *H.pluriseptata* (CCF 3788) under *in vitro* conditions. Colony morphology on PDA was comparable to that observed on CMD, MLA, OA, PCA, and PCASW, exhibiting abundant aerial mycelium and reaching 40–52 mm diam. in 2 wk. In contrast, growth on PDA with added NaCl was significantly reduced, with colonies reaching only 6–9 mm in diam. in 2 wk. Hujslová et al. (2010) examined the effects of pH and salinity on the growth of CCF 3787 and CCF 3788 using soil agar supplemented with rose Bengal and glucose.

The multicellular brown conidia of *Halomyrma* resemble those of *Moromyces* (Abdel-Wahab et al. 2010), a genus that currently includes a single species, *M.varius*.

#### ﻿ Thalassodendromyces

Taxon classificationAnimaliaLulworthialesLulworthiaceae

Vohník & Réblová gen. nov.

9249A4AC-4EF1-5A59-9969-7988A588D582

MB857652

Figs 5

##### Etymology.

Derived from *Thalassodendron*, the genus of seagrass from which the fungus was isolated, and the Greek word *mykes* (fungus). Referring to the association of the fungus with the seagrass *Thalassodendron*.

##### Type species.

*Thalassodendromycespurpureus* Vohník & Réblová.

##### Description.

**Asexual morph.** Observed exclusively in culture. Conidiophores, conidiogenous cells, and conidia are absent. **Mycelium** composed of cylindrical, hyaline, subhyaline, or brown, septate, branched, often slightly swollen to monilioid hyphae. **Monilioid hyphae** develop either terminally or laterally, solitary or in clusters. They are composed of thick-walled, globose to subglobose cells that mature gradually, starting as hyaline and becoming dark brown or dark olivaceous brown with age. **Sexual morph.** Not observed.

#### ﻿ Thalassodendromyces purpureus

Taxon classificationAnimaliaLulworthialesLulworthiaceae

Vohník & Réblová sp. nov.

AB180642-B3CD-5067-8174-0617B8CBB645

MB858635

Figs 6 , 7

##### Etymology.

From Latin *purpureus* (purple, violet, dark red). Referring to the colour of the pigment produced in culture.

##### Type.

MAURITIUS • northeast coast of Mauritius near Plage des Vignots; 20°06.98'S, 57°45.17'E; at a depth of 0.5–1.5 m; isolated from surface-sterilised roots of the seagrass *Thalassodendronciliatum*; 7 Dec 2023; leg. O. Hynar, isol. M. Vohník MAU-7 (**holotype**PRA-22363 dried culture on PCASW, ex-type culture CBS 153577 = CCF 6837, **paratype**PRA-22364 dried culture on MLA). GenBank: ITS = PV441107, LSU = PV441121, SSU = PV441114.

##### Culture characteristics.

On MLA colonies 11–15 mm diam. in 8 wk, circular, convex, margin ranging from entire to rhizoidal. The texture varied from mucoid to waxy, cobwebby at the centre and on the inoculation block, surface initially rugose, becoming cerebriform, and gradually developing deep radial furrows and cracks. Colonies were cream-coloured to yellow-beige, later exhibiting a deep purple pigment diffusing into the agar at the centre and margin, as well as forming irregular spots, often distinctly zonate. Over time, the colonies become purple to purple-brown, with submerged purple hyphae at the margins, reverse dark ochre with creamy or purple margin. On PCA colonies 17–20 mm diam. in 8 wk, circular to slightly irregular, flat, margin rhizoidal, cobwebby to mucoid, chestnut, ochre at the margin, with a conspicuous submerged growth, reverse of the same colour. On PCASW colonies 70–98 mm diam. in 2 wk. Colonies were initially composed of submerged mycelium, formed by sparse white vegetative hyphae visible only in translucent light (1 wk); later, hyphae became denser and subhyaline (2 wk) and gradually darkly pigmented (3 wk). In 8 wk, colonies were circular, flat, margin rhizoidal, dark brown, whitish grey at the centre, dark grey to almost black towards the margin; the texture ranged from cottony to sparsely floccose at the centre, cobwebby towards the margins, occasionally exhibiting zonation with alternating zones of sparse and dense growth, conspicuous submerged growth, with numerous colourless exudates on aerial hyphae, reverse none. Sporulation absent on MLA, PCA, and PCASW, except for the presence of monilioid hyphae (see description).

**Figure 6.**
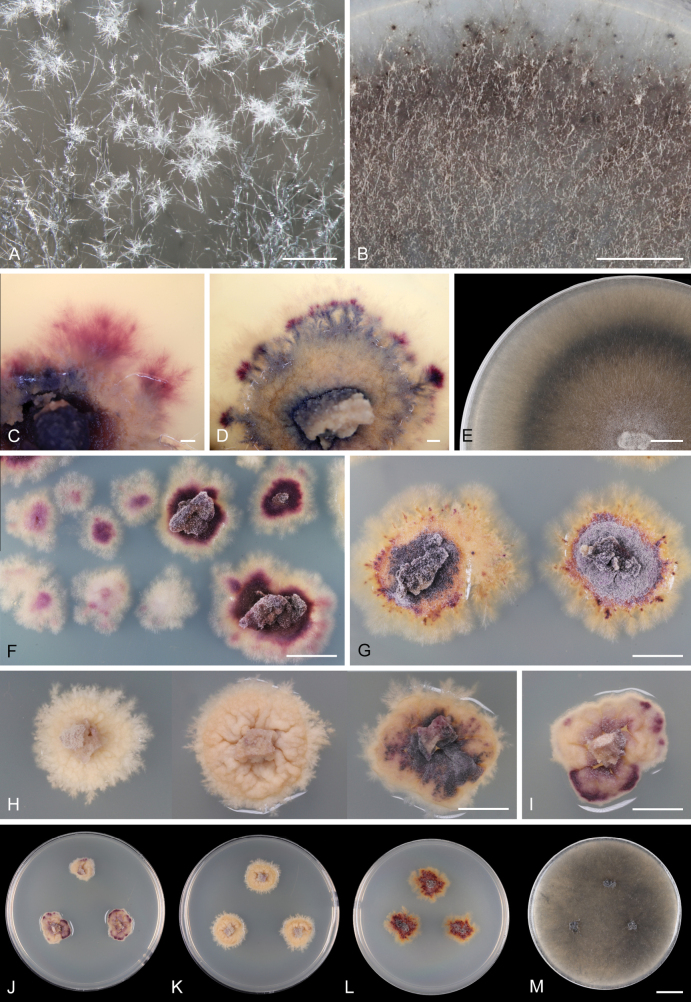
*Thalassodendromycespurpureus*. A, B, E. Colonies showing aerial hyphae, radial growth, and a zonation pattern of sparse and dense growth. C, D, F, G. Colonies showing purple pigmentation and distinct zonation patterns. H, I. Colonies exhibiting variability in morphology and pigmentation. J–M. Diversity of colony morphology on MLA, MLA, PCA, and PCASW, respectively (from left to right) after 8 wk. Images: on PCASW (A, B, E); on MLA (C, D, F–I). Strains: MAU-1 (A); CBS 153576 (I, J); CBS 153646 (C, H, K); CBS 153577 (D, G, L, M); CBS 153578 (E); PRA-22498 (B); CBS 153625 (F). Scale bars: 500 µm (A); 5 mm (B, E, F–I); 1 mm (C, D); 1 cm (J–M).

**Figure 7.**
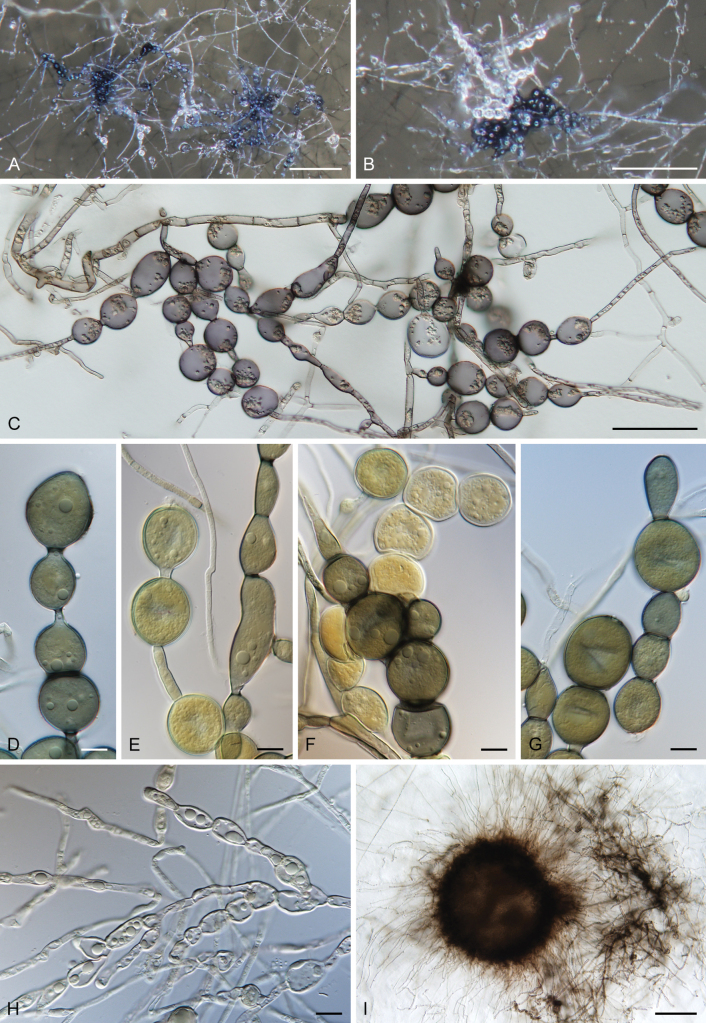
*Thalassodendromycespurpureus*. A, B. Clusters of dark-pigmented monilioid hyphae. C. Monilioid hyphae comprising thick-walled, globose to subglobose cells. D–G. Different stages of monilioid hyphae formation. H. Septate, hyaline vegetative hyphae with irregular swellings. I. Dense, dark, spherical aggregation of hyphae forming a compact structure in culture. Images: on PCASW (A–G, I); on MLA (H). Strains: CBS 153577 (A, B, D–G); CBS 153578 (H); CBS 153624 (C, I). Scale bars: 200 µm (A, B); 50 µm (C); 10 µm (D–H); 100 µm (I).

##### Description in culture.

**Asexual morph.** Conidiophores, conidiogenous cells, and conidia are absent. On PCASW, **mycelium** composed of hyphae 2–4 μm wide, cylindrical, septate, branched, hyaline at first, becoming subhyaline to brown; hyphae often slightly swollen, 5–7.5 μm wide, or monilioid. **Monilioid hyphae** develop either terminally or laterally, solitary or in clusters, which appear on the surface of colonies or immersed in agar. They are composed of thick-walled cells of different shapes: subglobose to globose 15–38 × 14–35 μm (mean ± SD = 23.6 ± 5.2 × 22 ± 4.6 μm), or ellipsoidal 15.5–38(–47) × 9–18 μm (mean ± SD = 26.2 ± 7.5 × 12.9 ± 3 μm), with walls 0.5–1 μm thick. The monilioid hyphae elongate on the terminal end; cells mature gradually, starting as hyaline and becoming dark brown to dark olivaceous brown with age, collapsing in old cultures (ca. 3 mo old). Sterile, globose structures developed at margin of the colonies; they are composed of dark brown, pseudoparenchymatous cells surrounded by sterile brown hyphae. On MLA, **mycelium** composed of hyphae 2–3 μm wide, cylindrical, septate, branched, hyaline, becoming dark brown in old cultures (ca. 4–6 mo old); hyphae often swollen, 3.5–5 μm wide, or monilioid. **Monilioid hyphae** occurring both on the surface of the colony and immersed in the agar. They are composed of subglobose to globose cells 11–17 × 9.5–15.5 μm (mean ± SD = 14.6 ± 2.0 × 12.2 ± 1.9 μm) and somewhat ellipsoidal cells 12.5–18 × 7.5–10 μm (mean ± SD = 15.2 ± 1.9 × 8.8 ± 0.9 μm), with walls 1–1.5 μm thick, hyaline. In old cultures (ca. 8 mo old), the cells in monilioid hyphae become dark brown. **Sexual morph.** Not observed.

##### Additional specimens examined.

MAURITIUS • northeast coast of Mauritius near Plage des Vignots; 20°06.98'S, 57°45.17'E; at a depth of 0.5–1.5 m; isolated from surface-sterilised roots of the seagrass *Thalassodendronciliatum*; 7 Dec 2023; leg. O. Hynar, isol. M. Vohník MAU-4 (**paratype**PRA-22365 dried culture, culture CBS 153576 = CCF 6836); • Ibid.; MAU-5 (**paratype**CBS H-25755, PRA-22509 dried culture, culture CBS 153646); • Ibid.; MAU-9 (**paratype**PRA-22367 dried culture, culture CBS 153578 = CCF 6838); • Ibid.; MAU-13 (**paratype**CBS H-25756 dried culture, culture CBS 153623); • Ibid.; MAU-16 (**paratype**CBS H-25757, PRA-22508 dried culture, culture CBS 153624); • Ibid.; MAU-19 (**paratype**CBS H-25758, PRA-22510 dried culture, culture CBS 153625); • Ibid.; MAU-17 (**paratype**PRA-22498 dried culture); • Ibid.; MAU-8 (**paratype**PRA-22366 dried culture); • Ibid.; MAU-1 (culture no longer viable). All dried cultures are on PCASW.

##### Habitat and geographical distribution.

The species is known as a root mycobiont of the seagrass *Thalassodendronciliatum* in Mauritius. No samples with identical sequences have been recorded in the GlobalFungi database.

##### Notes.

Clusters of dark monilioid hyphae composed of thick-walled, brown cells were only observed on PCASW (Fig. 7A–G). The dark, spherical aggregations of hyphae forming compact structures embedded within the agar medium remained sterile and may represent early stages of ascomatal formation (Fig. 7I). While monilioid hyphae were also present on MLA (Fig. 7H) and PCA, they mostly appeared as composed of irregularly swollen cells that remained hyaline, becoming pigmented in old cultures. Such cells never formed clusters and were reduced in both shape and size compared to those formed on PCASW.

On PCASW, the fungus developed extensive, dark, effuse colonies characterised by distinct submerged growth, sparse mycelium, and monilioid hyphae. Similar colonies were observed on PDA with NaCl. In contrast, on MLA and PCA lacking seawater, growth was considerably slower, and colonies appeared more compact with densely interwoven hyphae, displaying a mucoid-waxy texture. On MLA, the fungus produced a deep purple pigment. Growth on PDA and PDA supplemented with NaCl varied among the strains of *T.purpureus* (CBS 153576, CBS 153577, CBS 153578), and in contrast to *H.pluriseptata* (CCF 3788), was generally more vigorous on the salt-amended medium. The ex-type strain CBS 153577 exhibited the most robust growth, forming colonies 43–45 mm in diam. on PDA with NaCl within 2 wk, while growth on PDA alone was considerably slower, with colonies reaching only 6–7 mm in the same period. Strains CBS 153576 and CBS 153578 showed little to no growth on either medium, with mycelium developing only around the inoculation block.

*Thalassodendromycespurpureus* was the only fungus isolated from the roots of *T.ciliatum* that were heavily colonised by DS hyphae. This fact, together with similar hyphal morphology observed in the host and in culture (see the “Microscopic observations” in the Results), strongly suggests that the isolated fungus described here represents the mycobiont forming the newly described root-fungus symbiosis in *T.ciliatum*. However, a resynthesis experiment is necessary to confirm this in accordance with Koch’s postulates and to clarify the nature of the symbiosis, i.e. to determine where it lies along the mutualism–parasitism continuum.

## ﻿Discussion

### ﻿*Lulworthiales*: insights from molecular, cultivation, and environmental data

In marine ecosystems, the *Lulworthiales* are among the most dominant groups of filamentous fungi, yet their taxonomic delineation has remained challenging since the order’s establishment (Kohlmeyer et al. 2000). In particular, issues persist regarding the polyphyletic nature of the genera *Lindra* and *Lulworthia* (Kohlmeyer et al. 2000; Campbell 2005; Campbell et al. 2005; Azevedo et al. 2017; Dayarathne et al. 2025), the lack of molecular data for *Haloguignardiadecidua*, the type species of the genus (Cribb and Cribb 1956), limited know­ledge of the complete life history for most genera, the absence of protein-coding genes for the majority of members, as well as the unresolved phylogenetic placement of lulworthialean fungi in relation to *Spathulospora*. Over the past two decades, research employing molecular data has led to the discovery of several new genera, including the reassignment of taxa previously placed in non-lulworthialean genera, now resolved within the order. Notably, some of these new taxa are characterised exclusively by their asexual morphologies, which were previously rarely reported within this group (Abdel-Wahab et al. 2010; Azevedo et al. 2017; Li et al. 2023; Braconcini et al. 2024). These advancements have contributed to the improved taxonomic and nomenclatural stability of this prominent group of marine fungi.

Traditionally considered marine, members of the *Lulworthiales* have been primarily documented in seawater-influenced environments, such as decayed driftwood, submerged plants, sea foam, corals, and algal and seagrass hosts. However, recent discoveries in saline inland soils and other terrestrial niches (Hujslová et al. 2010; Li et al. 2019; Xiao et al. 2020; this study), including a single report of *Lulworthia* sp. isolated from wood submerged in a freshwater lake (Cai et al. 2002), challenge this marine exclusivity and raise important questions about their ecological plasticity, dispersal mechanisms, and adaptive strategies. Likewise, the diversity of lulworthialean marine and halophilic fungi, their adaptations to osmotically stressed environments, and their interactions with seagrasses and other coastal plants are poorly known. This study addresses these gaps by characterising two novel fungal genera within the *Lulworthiales*, i.e. *Halomyrma* from saline soils and *Thalassodendromyces* from seagrass roots, expanding the known ecological range of this order beyond strictly marine habitats.

Marine fungi, including those within the *Lulworthiales*, typically require specialised culture conditions replicating their natural saline habitats to display their distinctive morphological traits. Despite the absence of their wild type and clear diagnostic features in the natural environment, the two newly described species, *H.pluriseptata* and *T.purpureus*, exhibited distinctive traits under culture conditions, allowing for detailed morphological characterisation. We experimented with a variety of nutrient media, some of which were supplemented with salt and seawater, to assess the morphological variability of these fungi in culture. These efforts not only facilitated the identification of unique phenotypic markers but also underscored the importance of tailored cultivation techniques for the study of marine fungal biodiversity and systematics.

### ﻿*Halomyrmapluriseptata*: a lulworthialean fungus bridging aquatic and terrestrial habitats

The defining morphological characteristic of *H.pluriseptata* in culture is the production of dark pigmented, multicellular conidia with a pore at each cell, borne on holoblastic conidiogenous cells on aerial and submerged vegetative hyphae. Initially, young conidia consist of only a few cells separated by transverse septa. They begin as straight structures but gradually become tightly coiled. As they mature, a few longitudinal septa may form, and additional cells develop in multiple planes, resulting in a dense, interwoven mass of cells. This morphology aligns well with the concept of asexual morphs known in the *Lulworthiales*, many of which exhibit helicoid conidia. *Halomyrmapluriseptata* resembles *Moromyces* (Abdel-Wahab et al. 2010), a genus recently segregated from *Cumulospora* based on the presence of irregularly helicoid, transversely septate to muriform conidia. *Moromycesvarius* is a saprobic species occurring on decayed driftwood in the intertidal zone, with records from Egypt, Japan, and Thailand. Despite their superficial morphological similarities, phylogenetic analyses confirm that *Halomyrma* and *Moromyces* represent distinct evolutionary lineages.

In contrast to *T.purpureus*, *H.pluriseptata* displayed abundant growth and sporulation on all tested media. No notable differences were observed between PCASW and the other media devoid of salt, suggesting the organism’s ability to utilise diverse nutrient sources and adapt to varying osmotic pressures. Furthermore, the results from the geographical analysis confirm these observations and indicate that its occurrence is not strictly dependent on saline habitats.

*Halomyrmapluriseptata* was isolated from slightly saline soils in Czechia. While eDNA metabarcoding studies have confirmed the presence of the *Lulworthiales* within fungal soil communities in coastal saline ecosystems, such as unflooded soils, tidal flats, and coastal marshes (Li et al. 2019; Xiao et al. 2020), their occurrence in inland saline habitats remains undocumented. Although this study represents the first morphological characterisation and description of *H.pluriseptata*, the species has already been detected anonymously through multiple eDNA metabarcoding studies across a wide range of aquatic and terrestrial biomes worldwide. This highlights the importance of morphological characterisation and the placement of cultivable fungi within the fungal system using molecular DNA data, as well as providing valuable reference sequences for future research.

The data retrieved from the GlobalFungi database indicate a strong preference of *H.pluriseptata* for aquatic biomes, particularly marine and estuarine, where it is commonly associated with sediments and plant roots. Occasionally, it was also detected in freshwater habitats. In contrast, occurrences in terrestrial biomes appear to be sporadic and mainly in soil-associated niches. The broad distribution across diverse biomes suggests that, while the fungus is primarily adapted to aquatic ecosystems, it demonstrates ecological plasticity, allowing it to persist in select terrestrial habitats, possibly through dispersal mechanisms or adaptive traits suited for soil environments. It also highlights its ecological adaptability to various climate regions, suggesting that *H.pluriseptata* is not confined to a specific climatic range but can persist in both warm and cooler environments. Its distribution is likely influenced by habitat characteristics such as salinity, substrate type, and plant associations.

The species was initially classified as *Lulwoana* sp. based on a comparison of its partial SSU sequence, which showed 98% similarity to *Lu.uniseptata*CBS 167.60 (AY879034; Hujslová et al. 2010). While the relatively conserved 18S rDNA gene is useful for identifying organisms at the genus or higher taxonomic levels, accurate interpretation of similarity percentages is essential (Wu et al. 2015; Chittavichai et al. 2025). In fact, the observed sequence similarity suggests that the soil-derived fungus may represent a novel genus related to *Lulwoana* within the *Lulworthiales*, a placement that was confirmed by our three-gene phylogenetic analysis. In this study, *Lulwoana* (Campbell et al. 2005), typified by *Lu.uniseptata*, is treated as a synonym of *Zalerion* based on *Z.nepura* (Moore and Meyers 1962), which is in line with the typification provided by Gonçalves et al. (2021). Anastasiou (1963) synonymised four *Zalerion* species, including *Z.nepura*, under *Z.maritima*, now the type species of the genus. Nakagiri (1984) experimentally demonstrated the conspecificity of the sexual *Lu.uniseptata* and asexual *Z.maritima*. The ex-type strain of *Lu.uniseptata* (NBRC 32137) forms a monophyletic clade with another isolate of *Z.maritima* (Azevedo et al. 2017) and *Z.pseudomaritima* (Gonçalves et al. 2021). Nonetheless, Dayarathne et al. (2025) dismissed these findings, contending that *Zalerion* is polyphyletic and proposing the continued use of *Lulwoana*.

### ﻿Morphology and phylogenetic distinctiveness of *Thalassodendromycespurpureus*

While genera within the *Lulworthiales* are well-characterised based on morphological and phylogenetic features, and the majority of them were collected on decaying organic matter, the nature of their interactions with plants and small marine animals—whether saprobic, endophytic, or parasitic—remains insufficiently understood and requires further investigation. This is particularly illustrated by introducing the third known root-fungus symbiosis in seagrasses, between *T.ciliatum* and *T.purpureus*. The need to explore additional habitats beyond those traditionally associated with primarily endophytic fungi is highlighted by the study of Lefebvre et al. (2023), who investigated the marine fungus *Pos.atricolor* (*Pleosporales*) (Vohník et al. 2019), initially isolated as a DS endophyte from the roots of the dominant Mediterranean seagrass *P.oceanica*. Lefebvre et al. (2023) documented that saprobic activity of *Pos.atricolor* contributes to the progressive degradation of *P.oceanica* tissues, beginning in living plants and persisting through the matte (an organo-mineral sediment formed by the seagrass) and potentially into the leaf litter. These authors suggested that the *Pos.atricolor* saprobic lifestyle extends past its typical endophytic association with its seagrass host.

In this study, *T.purpureus* was the only mycobiont isolated from the roots of the seagrass *T.ciliatum* from Mauritius. The studied strains did not form typical sexual or asexual reproductive structures but instead produced monilioid hyphae, solitary or in clusters, that were exclusively observed on PCASW. It remains unclear whether these cells function as chlamydospores; the hyphae were not observed to fragment into solitary cells, and the cells tended to collapse with age. Nevertheless, these monilioid hyphae were reminiscent of chlamydospores described in *Halophilomyceshongkongensis* (Wang et al. 2024), *Lulworthiaatlantica* (Azevedo et al. 2017), several *Paralulworthia* species (Poli et al. 2020), and *Rambellisea* (Braconcini et al. 2024). In *Paralulworthia*, chlamydospores are occasionally also multicellular, arranged in tetrads or irregular shapes.

The close relationship between *Thalassodendromyces* and *Spathulospora* is particularly interesting from an ecological perspective, as members of *Spathulospora* are biotrophic parasites of the red macroalga *Ballia*, whereas *T.purpureus* manifests what appears as an endophytic association within the roots of the seagrass *T.ciliatum*. In a narrow sense, endophytes live asymptomatically in plant tissues, i.e. without causing apparent benefit or damage to the host plant (Wilson 1995). Living isolates of *Spathulospora* species are not available, and their cultivation in axenic culture may be particularly challenging due to their parasitic lifestyle. Although the asexual morph of *Spathulospora* remains unknown, preventing direct comparison with related taxa apart from sexual characters, its species are characterised by the formation of conspicuous trichogynes and antheridia (Kohlmeyer 1973). Antheridia exhibit a penicillate branching pattern or a more compact arrangement. They consist of a stalk and a penicillately branched head composed of multiple supporting cells or branches, each carrying one to several lageniform, phialide-like antheridia with a tubular neck.

In contrast, two other known seagrass endophytes in the *Lulworthiales*, namely *Paralulworthia* (Poli et al. 2020, 2021) and the invalidly published genus *Halophilomyces* (Wang et al. 2024), showed no close phylogenetic relationship to *Thalassodendromyces*. Apart from *P.halima*, which was collected from submerged wood, other *Paralulworthia* species have been isolated from the roots and rhizomes of *P.oceanica*, while *H.hongkongensis* was recovered from the roots and rhizomes of *Halophilaovalis*. However, their potential symbiotic activity has never been examined experimentally.

*Thalassodendromycespurpureus* is so far known only from the roots of *T.ciliatum* from one locality in Mauritius, a situation similar to *Pos.atricolor*, which is only known as an associate of *P.oceanica* (Vohník et al. 2019; Frasca et al. 2024a, 2024b).

### ﻿Taxonomic challenges and molecular and morphological perspectives on *Spathulosporales*

Given the parasitic lifestyle of *Spathulospora*, the simplified thallus without true hyphae, the ascospore morphology, and the thin-walled, filamentous trichogynes and phialide-like antheridia in its various species, Kohlmeyer (1973) established the monotypic order *Spathulosporales*. This order was characterised by dark, crustose, irregular, thick-walled cells covering the host, which is penetrated by a peg-like cell. Additional defining features include perithecial ascomata developing on a crustose subiculum, the absence of paraphyses, pseudoparenchymatous ascogenous hyphae, and unitunicate asci that deliquesce early, each containing eight one-celled, hyaline ascospores with spatulate ends due to lateral appressed appendages. Members of the order *Spathulosporales*, which comprise marine fungi parasitic on red algae, include three genera: *Spathulospora (Spathulosporaceae)* and *Hispidicarpomyces* and *Retrostium (Hispidicarpomycetaceae)* (Nakagiri 1993; Nakagiri and Ito 1997).

Inderbitzin et al. (2004) analysed *Spathulospora* based on LSU and SSU rDNA sequences obtained from two herbarium specimens: an air-dried sample of *S.adelpha* (J.K. 5599, GenBank: AY380314) and a specimen preserved in formalin of *S.antarctica* (J.K. 3530, AY380315). Molecular DNA data provided evidence that *Spathulospora* is nested within the *Lulworthiales*, where it represents a terminal branch distinguished by its parasitic lifestyle and a body structure adapted to this mode of life. This sets it apart from other members of the order, which typically exhibit hyphal thalli and are primarily saprobic or endophytic. To date, *Spathulospora* has not shown a well-supported affiliation with any recognised genera within the *Lulworthiales*. It has consistently been placed within a broader clade alongside *Cumulosporamarina*, *Kohlmeyeriella* spp., *Lindriella*, and *Sammeyersia* (Inderbitzin et al. 2004; Campbell et al. 2009; Abdel-Wahab et al. 2010, 2017). However, aside from BI analyses, this relationship has not been statistically supported in maximum parsimony, ML, or neighbour-joining analyses. In Dayarathne et al. (2025), the relationship between *Spathulospora* and *Cumulospora*–*Lindriella* clades was supported exclusively by the ML analysis. In our study, *Spathulospora* and *Thalassodendromyces* exhibited a statistically strongly supported relationship. Additionally, the node comprising the clades of *Spathulospora*–*Thalassodendromyces* and *Cumulospora*–*Lindriella* was well supported in both BI and ML analyses.

The phylogeny and taxonomy of *Spathulosporales* remain unresolved due to the lack of living cultures and molecular data for *Hispidicarpomyces*, *Retrostium*, and *Spathulosporaphycophila*, the generic type (Cavaliere and Johnson 1965). Further research is essential to clarify their evolutionary relationships and to determine whether *Spathulosporales* should be retained as a distinct order or merged with the *Lulworthiales*. Except for the distinct antheridia and trichogynes, typically spatulate ascospores, and the non-hyphal thalli of *Spathulospora*—which are likely adaptations to the simple thallus of the red alga host *Ballia* (Nakagiri 1993)—other characteristics such as ascomata, hamathecium, and asci are largely comparable to those of other lulworthialean fungi.

### ﻿Salinity as a possible driver of fungal distribution

Exploring the fungal communities in saline environments can offer valuable insights into their survival strategies and help to clarify whether salinity is the primary factor shaping their distribution. Research on fungi inhabiting marine and terrestrial saline environments has revealed that halotolerant and extremotolerant fungi have evolved in various lineages of mainly *Ascomycota*, particularly the filamentous fungi and black yeast-like forms in *Chaetothyriales* and *Eurotiales (Eurotiomycetes)*, as well as in *Capnodiales*, *Cladosporiales*, *Dothideales*, and *Pleosporales (Dothideomycetes)*, and *Basidiomycota*, i.e. *Wallemiales (Wallemiomycetes)*. Genera such as *Aspergillus*, *Aureobasidium*, *Cladosporium*, *Cladophialophora*, *Exophiala*, *Hortaea*, and *Wallemia*, among others, have frequently been recovered from a range of saline habitats, including coastal solar salterns, coastal rocks, coral reefs, hypersaline water bodies, marine sponges, saline desert crusts, saline soils, salt marshes, and even salted food (e.g. Cantrell et al. 2006, 2011; Zalar et al. 2007; Kralj Kuncic 2010; Gostinčar et al. 2011; Gunde-Cimerman et al. 2018; Chung et al. 2019; Samson et al. 2019). Among these fungi, *Hortaeawerneckii* is a model halophilic, black yeast-like species that exhibits remarkable adaptability to saline environments. Its genome displays extensive gene duplication, likely associated with salt stress responses and specialisation for salt tolerance (Lenassi et al. 2013).

The taxonomic overlap observed among fungi inhabiting both marine and terrestrial saline environments supports the view that salinity acts as a dominant selective pressure. These fungi have developed convergent adaptive strategies, such as melanin production, osmolyte synthesis, and reduced growth rates, that enable them to persist across a range of salinity gradients. The data presented here on the occurrence of *H.pluriseptata* in both marine and terrestrial saline habitats provides one more example of halophilic adaptation across *Ascomycota*. Moreover, the presence of *Lulworthiales*, including *Halomyrma*, in continental saline soils suggests that salinity, rather than ecosystem type, is a key factor in defining the ecological niches of lulworthialean fungi.

## ﻿Conclusion

This study presents two novel fungal genera within the *Lulworthiales*, namely *Halomyrma* and *Thalassodendromyces*, expanding the known ecological range of this order from marine to terrestrial saline habitats. *Thalassodendromycespurpureus* is introduced as a previously unrecognised root-associated mycobiont of the seagrass *Thalassodendronciliatum* growing in coastal waters off Mauritius. This discovery represents the third documented root-fungus symbiosis in seagrasses, contributing to a broader understanding of seagrass–fungal interactions and their ecological significance in marine ecosystems. In contrast, *Halomyrmapluriseptata* was isolated from saline inland soils in Czechia. Both genera form separate lineages within the *Lulworthiales*.

The detection of *H.pluriseptata* in marine, estuarine, freshwater, and even terrestrial ecosystems through eDNA metabarcoding underscores its ecological adaptability. Conversely, the lack of samples with sequences identical to those of *T.purpureus* in the GlobalFungi database indicates a restricted distribution of this species. This study further demonstrates the power of combining culture-based approaches with molecular phylogenetics and eDNA metabarcoding to resolve fungal diversity, distribution patterns, and biogeography at the global scale.

Given the phylogenetic position of the *Spathulosporales* and the lack of living isolates for its key taxa, future sequencing efforts on fresh material and cultivation studies will be essential to refine the systematics of the *Lulworthiales*, including its potential integration in *Spathulosporales*.

Our findings indicate that fungal diversity and ecological adaptations of the lulworthialean fungi remain underexplored, especially in habitats outside their historically recognised niches, including the mycobiomes of seagrasses and terrestrial biomes, which may harbour novel species with unique ecological roles.

## Supplementary Material

XML Treatment for Halomyrma

XML Treatment for Halomyrma pluriseptata

XML Treatment for Thalassodendromyces

XML Treatment for Thalassodendromyces purpureus

## ﻿Additional information

### Use of AI

The AI-generated figure in the background of the phylogenetic tree was created with ChatGPT (OpenAI 2025, version Feb 2025, retrieved on 10 February 2025).

### Adherence to national and international regulations

All the fungal strains used in this study have been legally obtained, respecting the Convention on Biological Diversity (Rio Convention).

### Author ORCIDs

Martina Réblová https://orcid.org/0000-0001-5229-1709

Jana Nekvindová https://orcid.org/0000-0002-2861-5483

Martin Vohník https://orcid.org/0000-0002-8655-2708

### Data availability

All of the data that support the findings of this study are available in the main text or Supplementary Information.

## Supplementary materials

Supplementary material 1 Alignment of ITS–LSU–SSU sequences Data type txt Explanation note Alignment of ITS–LSU–SSU sequences of members of the *Lulworthiales*. https://binary.pensoft.net/file/1393007This dataset is made available under the Open Database License (http://opendatacommons.org/licenses/odbl/1.0/). The Open Database License (ODbL) is a license agreement intended to allow users to freely share, modify, and use this Dataset while maintaining this same freedom for others, provided that the original source and author(s) are credited.Martina Réblová

Supplementary material 2 Supplementary table Data type xlsx Explanation note Biogeographical distribution, sample type, habitat, and other detailed metadata for *Halomyrmapluriseptata* inferred from the GlobalFungi database. https://binary.pensoft.net/file/1393008This dataset is made available under the Open Database License (http://opendatacommons.org/licenses/odbl/1.0/). The Open Database License (ODbL) is a license agreement intended to allow users to freely share, modify, and use this Dataset while maintaining this same freedom for others, provided that the original source and author(s) are credited.Martina Réblová
